# Supplementary material for: In silico docking yields small molecule negative allosteric modulators targeting the core of Frizzled 7
Source: Nat Commun. 2025 Dec 14;16:11138. doi: 10.1038/s41467-025-67147-z (PMC12705740; doi:10.1038/s41467-025-67147-z)
Supplement: Supplementary file 1 — Supplementary Information [file 41467_2025_67147_MOESM1_ESM.pdf]

In silico docking yields small molecule negative allosteric modulators targeting the core of Frizzled 7

Supplementary Information

Magdalena M. Scharf[1] Julia Kinsolving[1,#] Lukas Grätz[1,5,#] Jan Hendrik Voss[1]

David Carrasco-Busturia[2] Björn Forsberg[3] Peter Kolb[4] Gunnar Schulte[1,*]

[1] Department of Physiology & Pharmacology, Sec Receptor Biology & Signaling, Biomedicum, S-171 65 Stockholm, Sweden

[2] Division of Theoretical Chemistry and Biology, School of Engineering Sciences in Chemistry, Biotechnology and Health, KTH Royal Institute of Technology, SE-100 44 Stockholm, Sweden

[3] Department of Physics, Chemistry and Biology, SciLifeLab, Linköping University, Linköping 58 183, Sweden

[4] Philipps-Universität Marburg, Institute of Pharmaceutical Chemistry, Marburg, Germany

[5] Current address: Molecular, Cellular and Pharmacobiology Section, Institute of Pharmaceutical Biology, University of Bonn, 53115 Bonn, Germany

# Contributed equally.

*To whom correspondence should be addressed: gunnar.schulte@ki.se

Supplementary Figures

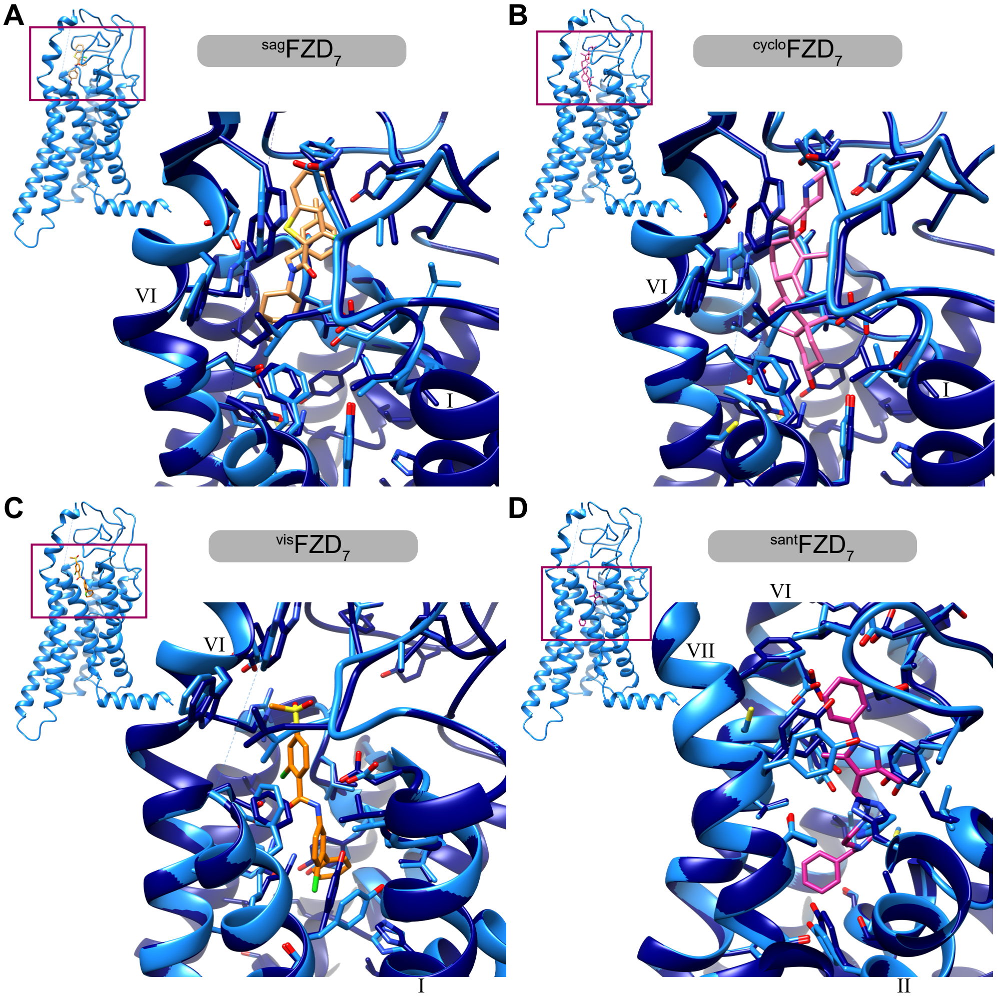

Supplementary Figure 1: Overview of the prepared docking setups for the initial screen. A: $^{sag}FZD_7$, B: $^{cyclo}FZD_7$, C:$^{sant}FZD_7$, D: $^{vis}FZD_7$. Ligands from SMO structures were merged into the FZD_7 structure after alignment. The molecules and residues in 5 Å distance around the molecule were then energy minimised to adapt the artificial binding pocket to binding of a ligand. The side-view of the receptor shows the approximate location of molecule/binding pocket in the 7TMD. Close-up show the comparison of the residues in 5 Å distance around the inserted molecule in the orignal structure 7EVW (dark blue) and after minimising together with the inserted molecule (dodger blue). The respective inserted molecule is highlighted in a different colour (sand: SAG1.5; pink: cyclopamine; orange: vismodegib; violet red: SANT-1). Numbering of the TMs is indicated in roman numerals.

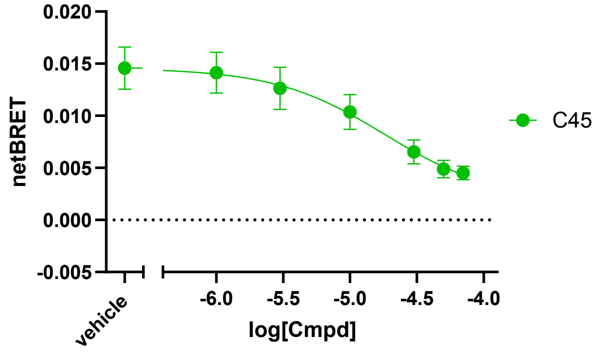

Supplementary Figure 2: Concentration response curve from BRET-based competition binding experiments with C45 and BODIPY-cyclopamine (Nluc-FZD_7). Datapoints are mean ± SEM of 5 independent experiments. Source data are provided as a Source Data file.

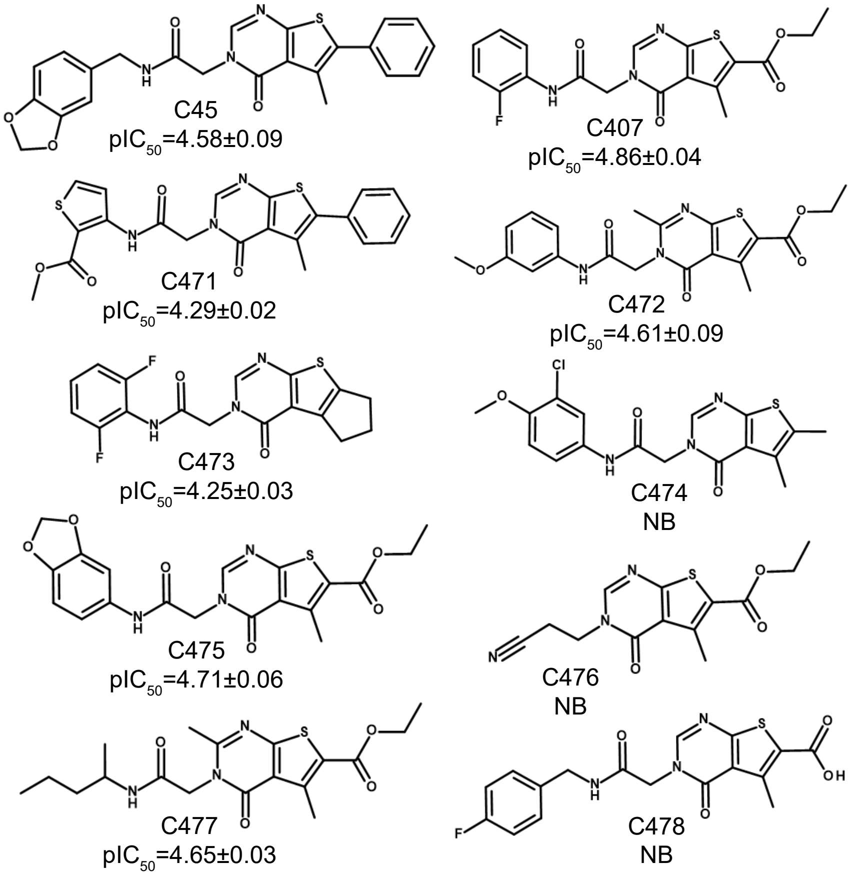

Supplementary Figure 3: Chemical structures and pIC_{50} values from competition binding experiments for hit molecules C45 and C407 as well as all compounds based on C407 (C471-C478). pIC_{50} are mean ± SEM for 3-5 independent experiments. NB: Not binding.

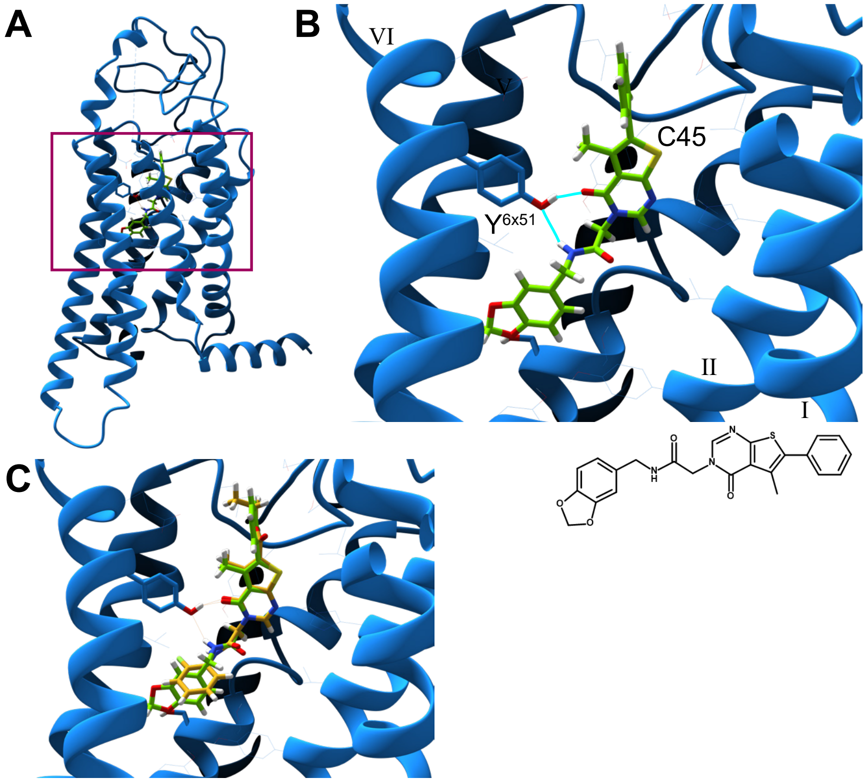

Supplementary Figure 4: Predicted binding pose of C45 from the docking calculations. A: Side view of the receptor. C45 (bright green) is predicted to bind deep within the 7TMD of FZD_7 (blue), as highlighted by the purple frame. B: Closeup of the predicted binding pose of C45 (bright green) within the receptor core. Compound C45 is predicted to form polar interactions with $Y489^{6x51}$, as indicated by bright blue lines. The chemical structure of C45 is depicted beneath the docking pose for reference. Numbering of the TMs is indicated in roman numerals. C: Overlay of the predicted binding poses of C45 (bright green) and C407 (goldenrod). Both compounds are predicted to bind in a similar pose. B,C: Note that TMs in the front are hidden for better visibility of the compound pose.

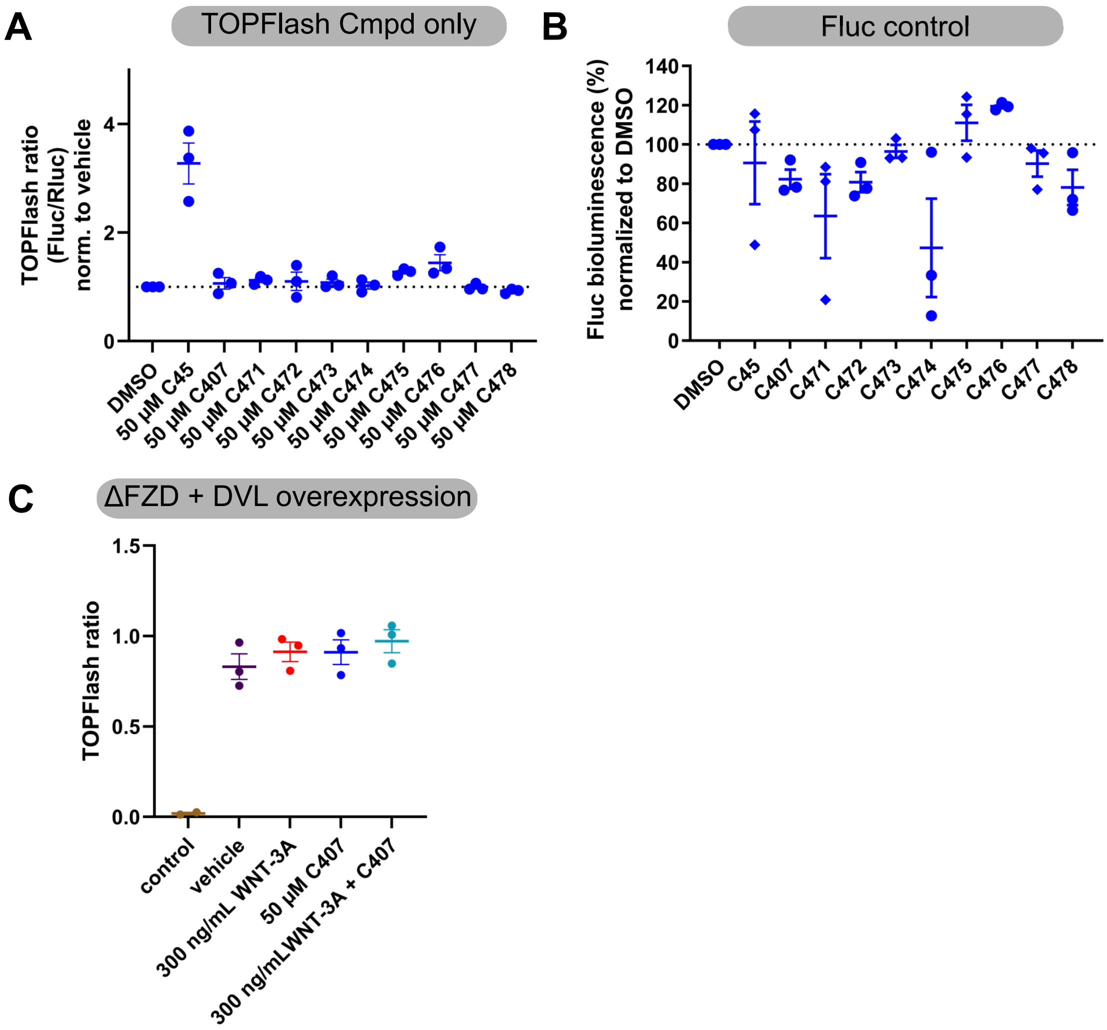

Supplementary Figure 5: Control experiments for the TOPFlash assay. A: TOPFlash responses induced by the compounds alone at HiBiT-FZD_7, which was transfected into $\Delta FZD_{1\text{-}10}$ HEK293T cells (no WNT stimulation) B: Control assay to exclude compound-mediated interference with Fluc bioluminescence. Experiments were performed in $\Delta FZD_{1\text{-}10}$ HEK293T cells constitutively expressing Fluc. C: Effect of WNT-3A and C407 stimulation on TOPFlash response induced by overexpression of DVL2 in $\Delta FZD_{1\text{-}10}$ HEK293T cells. Data points represent means ± SEM of three independent measurements performed in triplicates. Source data are provided as a Source Data file.

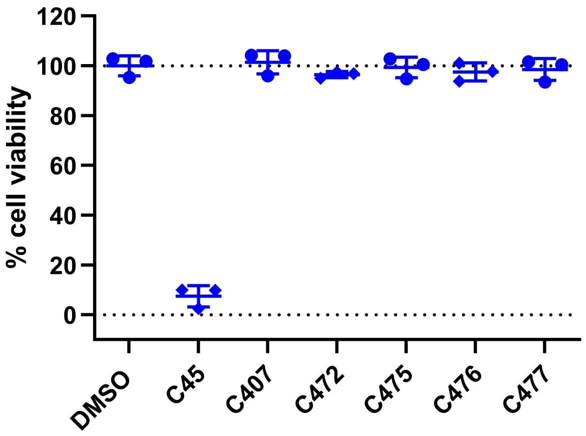

Supplementary Figure 6: Effect of selected compounds on cell viability of HEK293 cells. Cells were incubated with 10 µM of compound. Data points represent means ± SD of three independent measurements. Source data are provided as a Source Data file.

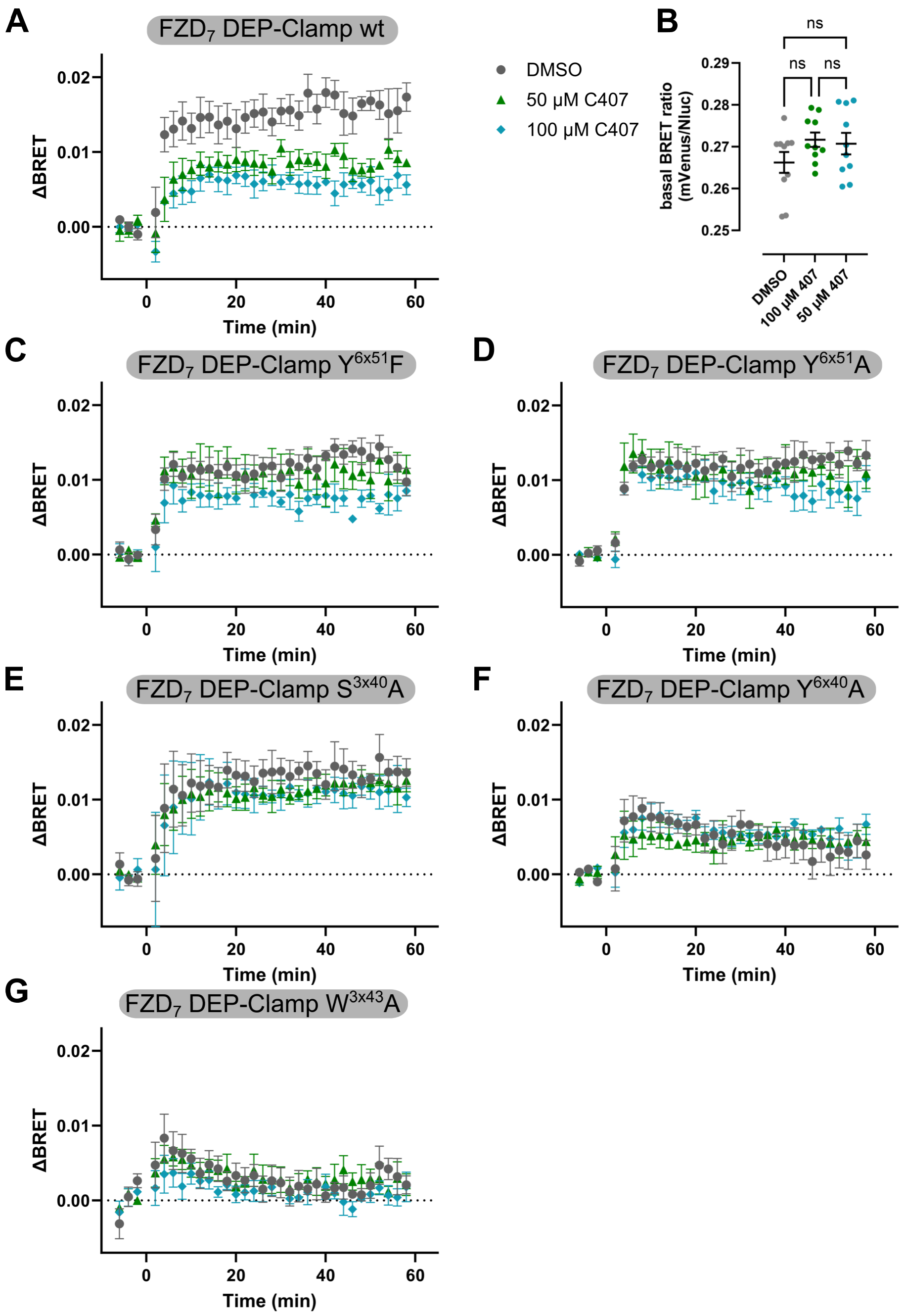

Supplementary Figure 7: Measurements of BRET changes in the FZD_7-DEP-Clamp sensor. Time-course measurements upon stimulation with WNT-3A only (DMSO; grey circles) and WNT-3A stimulation after pre-incubation with C407 (50 µM, green triangles, or 100 µM, blue squares) are shown for A: wild-type FZD_7; C: FZD_7 $Y489^{6x51}F$; D: FZD_7 $Y489^{6x51}A$; E: FZD_7 $S351^{3x40}A$; F: FZD_7 $Y478^{6x40}A$; and G: FZD_7 $W354^{3x43}A$. In all cases, 1 µg/mL WNT-3A was used. Data represent mean ± SEM for 3-5 independent experiments. B: Basal BRET values for the wild-type FZD_7-DEP-Clamp sensor after pre-incubation with DMSO or C407 only. Data are mean ± SEM from five independent experiments (technical replicates were averaged for each experiment). Source data are provided as a Source Data file.

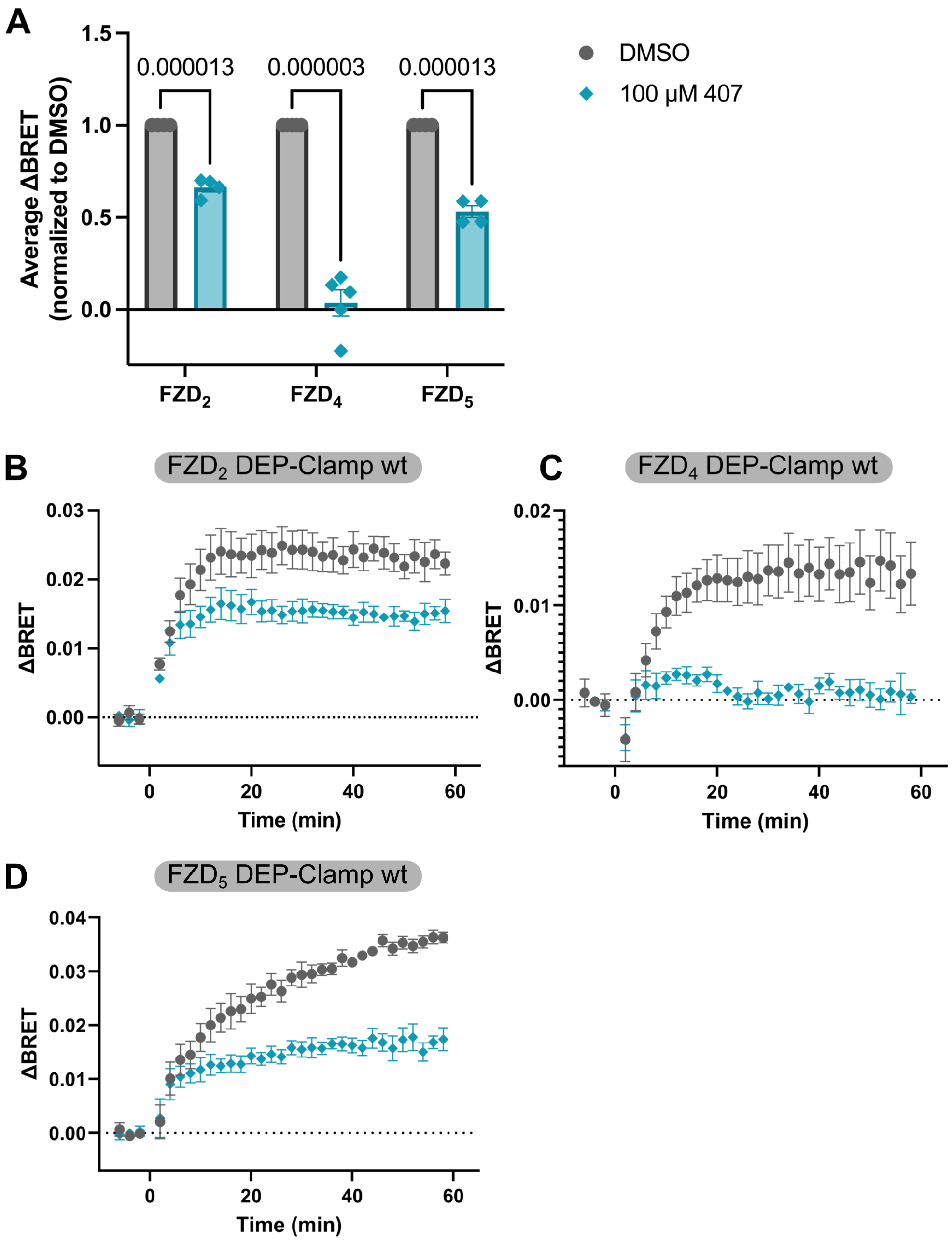

Supplementary Figure 8: Selectivity measurements of C407 as determined using the FZD_2-, FZD_4- and FZD_5-DEP-Clamp sensors. A: Bar charts showing the average BRET change after stimulation with WNT-3A (1 µg/mL) after pre-incubation with DMSO (grey circles) or C407 (100 µM, blue squares). B, C, D: Corresponding time-course measurements to A. Data are mean ± SEM for 4-5 independent experiments. Statistical analysis was performed with a multiple unpaired t-test. Source data are provided as a Source Data file.

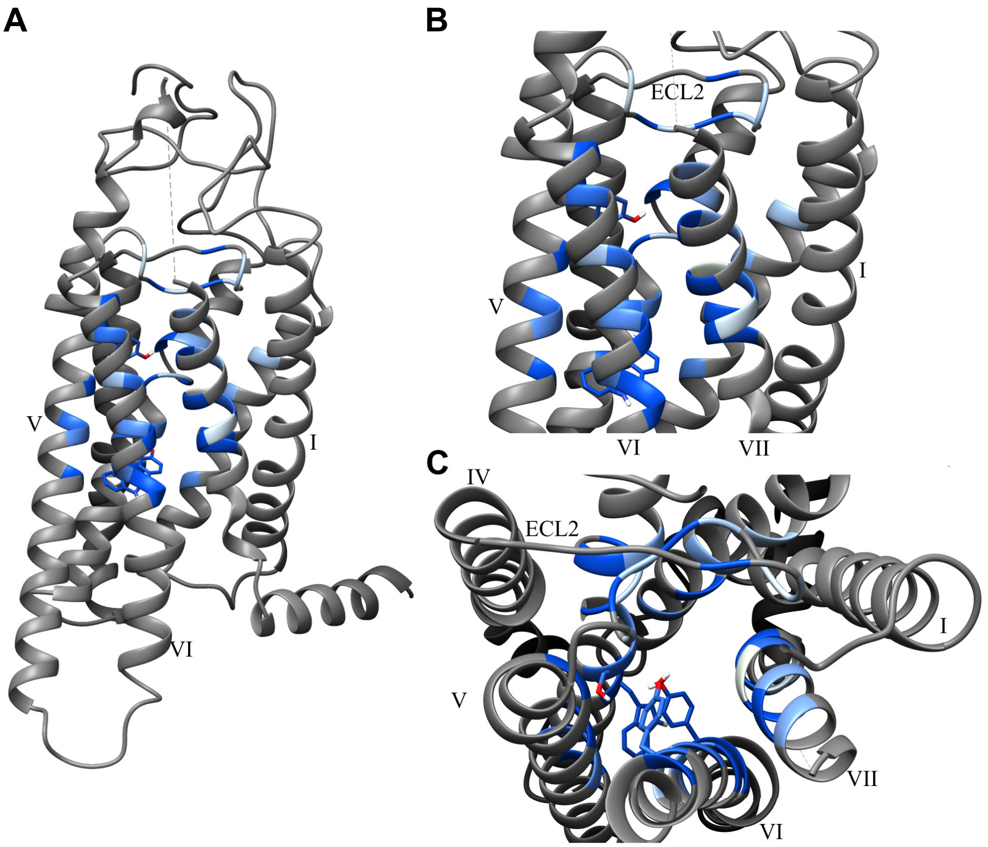

Supplementary Figure 9: Sequence conservation among all 10 FZD paralogs for residues within the potential binding site of C407 plotted on the FZD_7 structure as used for the docking calculations. A: Sideview of the receptor 7TMD. B: Same as A but with focus on the potential binding site region. C: Top view of the 7TMD. Residues within 5 Å distance around the docking poses of C407 and C45 as well as the poses of C407 as obtained in the two first clusters of both MD simulations were included in the analysis. A darker shade of blue indicates a higher conservation of the respective residue among all 10 FZD paralogs (lightest blue = 40 %; darkest blue = 100 %). Regions coloured in grey were not included in the analysis. Residues that were identified as relevant and mutated during the described study are shown as sticks ($S351^{3x40}$, $W354^{3x43}$, $Y478^{6x40}$and $Y489^{6x51}$). TM numbering is indicated in roman numerals. The plotted data can be found in Supplementary Table 1.

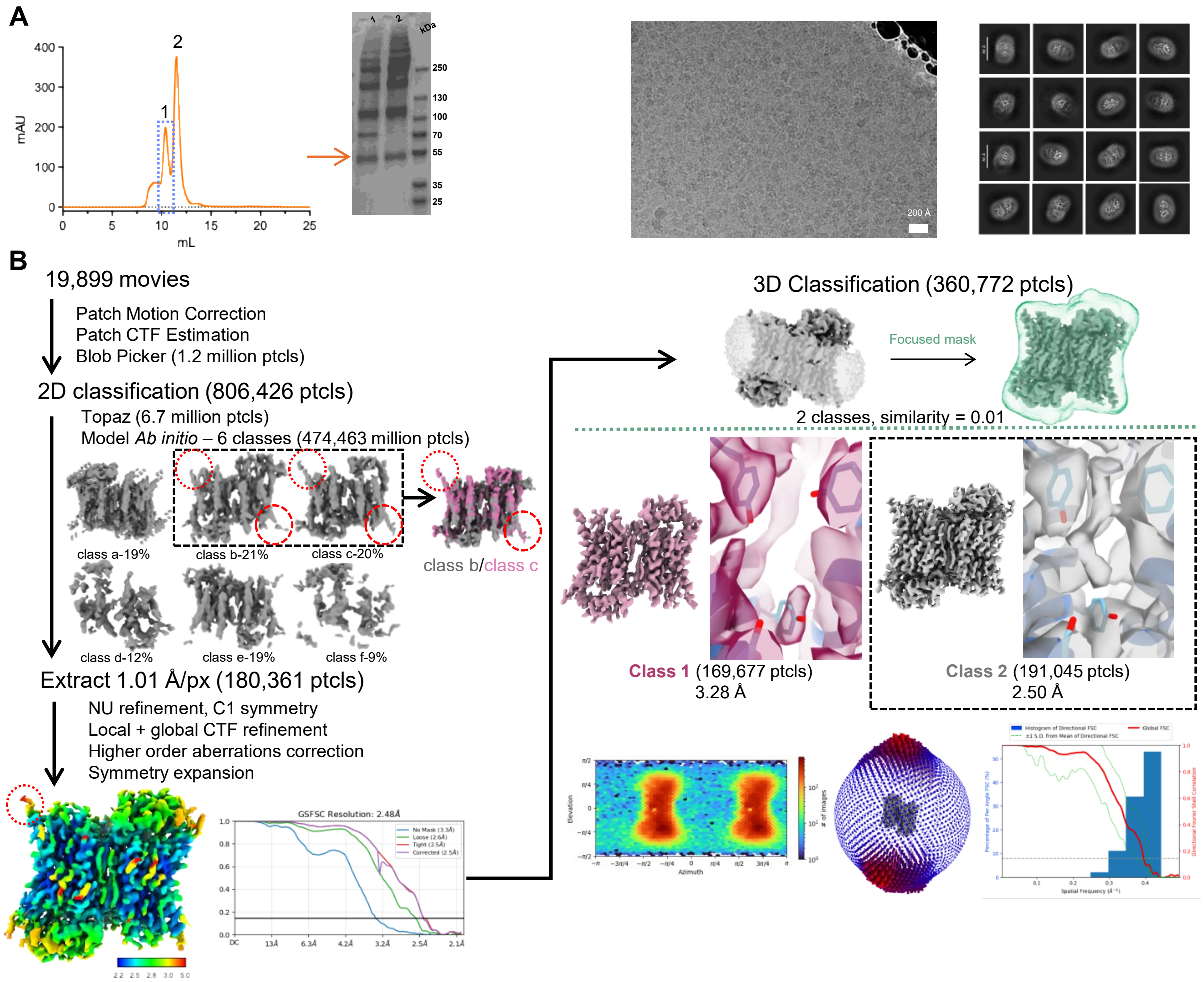

Supplementary Figure 10: Biochemical characterization and processing pipeline of FZD_7. A: Size exclusion chromatography (SEC) and Coomassie stain of purified FZD_7. The dashed line represents the fraction taken for cryo-EM analysis. A representative micrograph and 2D classifications are taken from the collection. B: The cryo-EM workflow for data processing is detailed. Azimuth plot, angular distribution of particles, and FSC curve for the final map are shown. See materials and methods for more details.

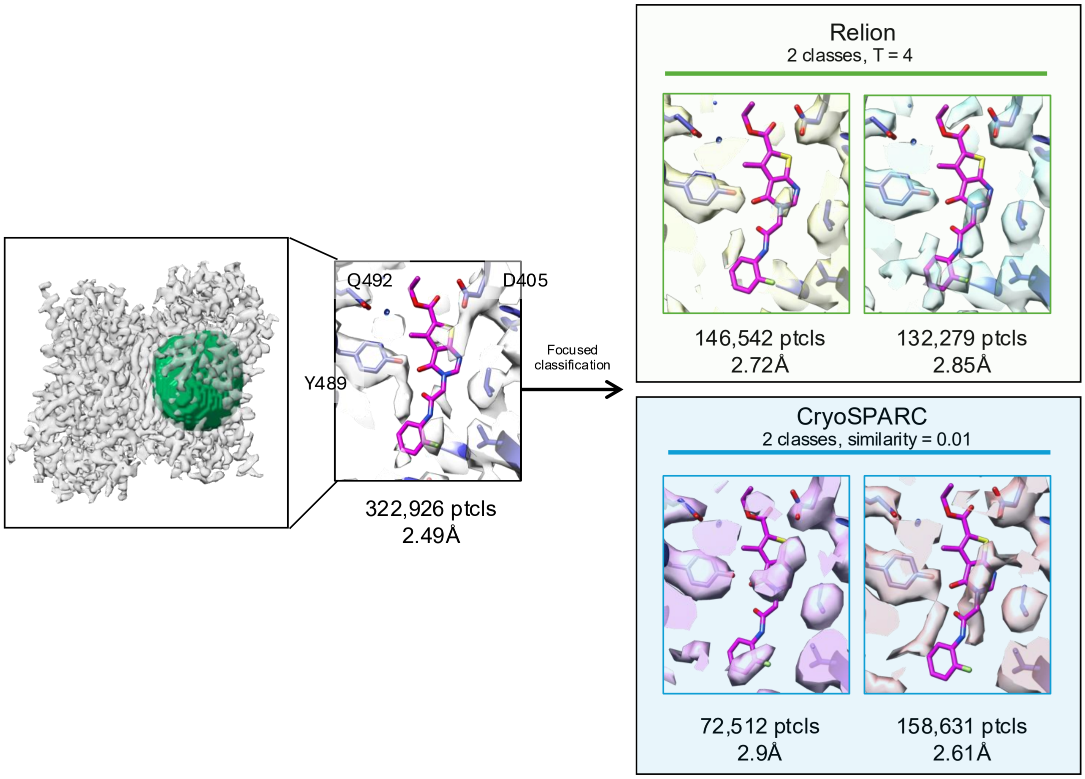

Supplementary Figure 11: Focused classification and local refinements of FZD_7. A smaller mask was generated for the region of interest in protomer B corresponding to the 7TMD and used for subsequent local refinements yielding indiscriminate classes. See materials and methods for more details.

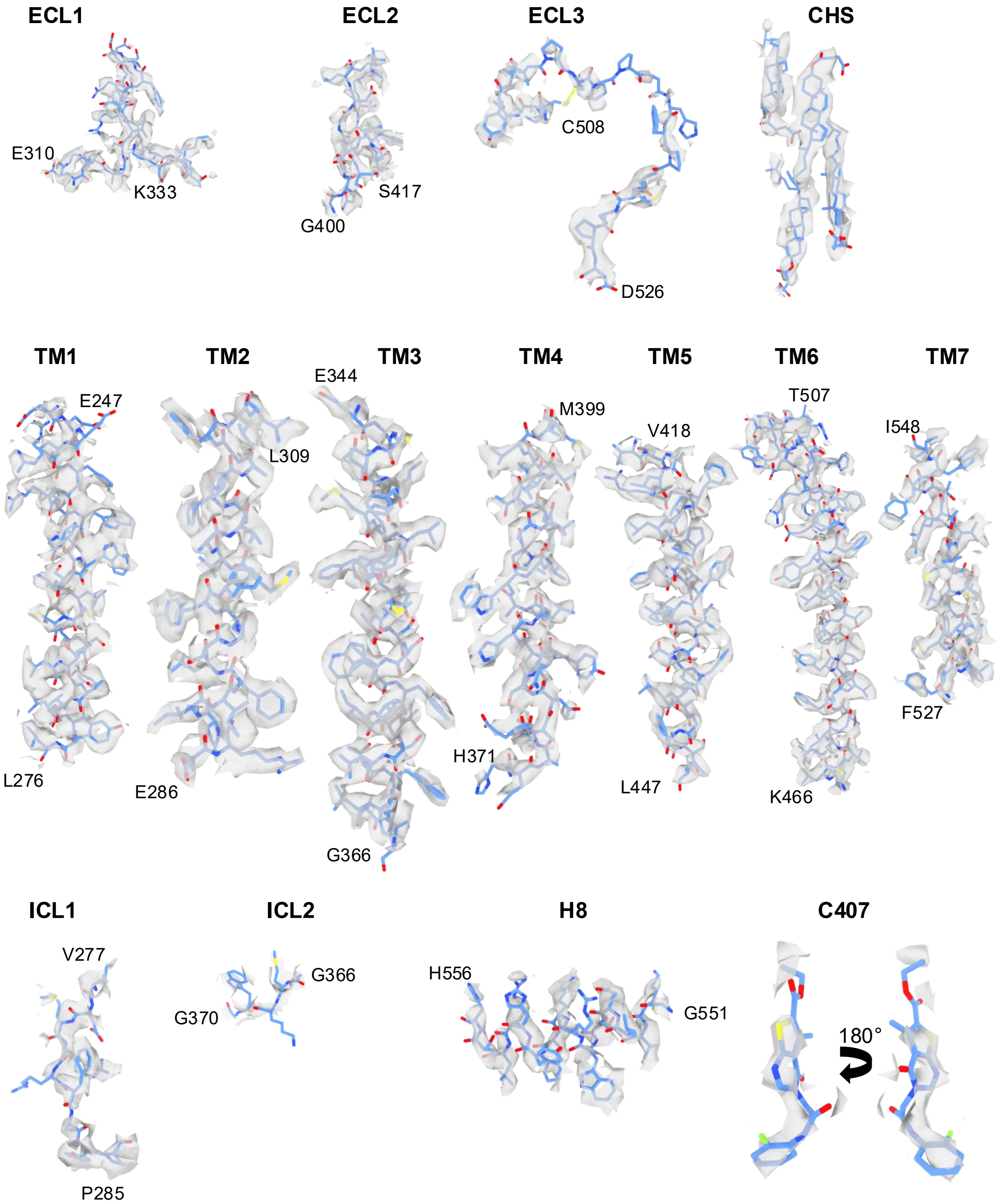

Supplementary Figure 12: Cryo-EM maps with the model fit of FZD_7. The density map of FZD_7 is shown with a surface representation (grey) and the contoured map as sticks (blue) for each respective domain, region, molecule or lipid.

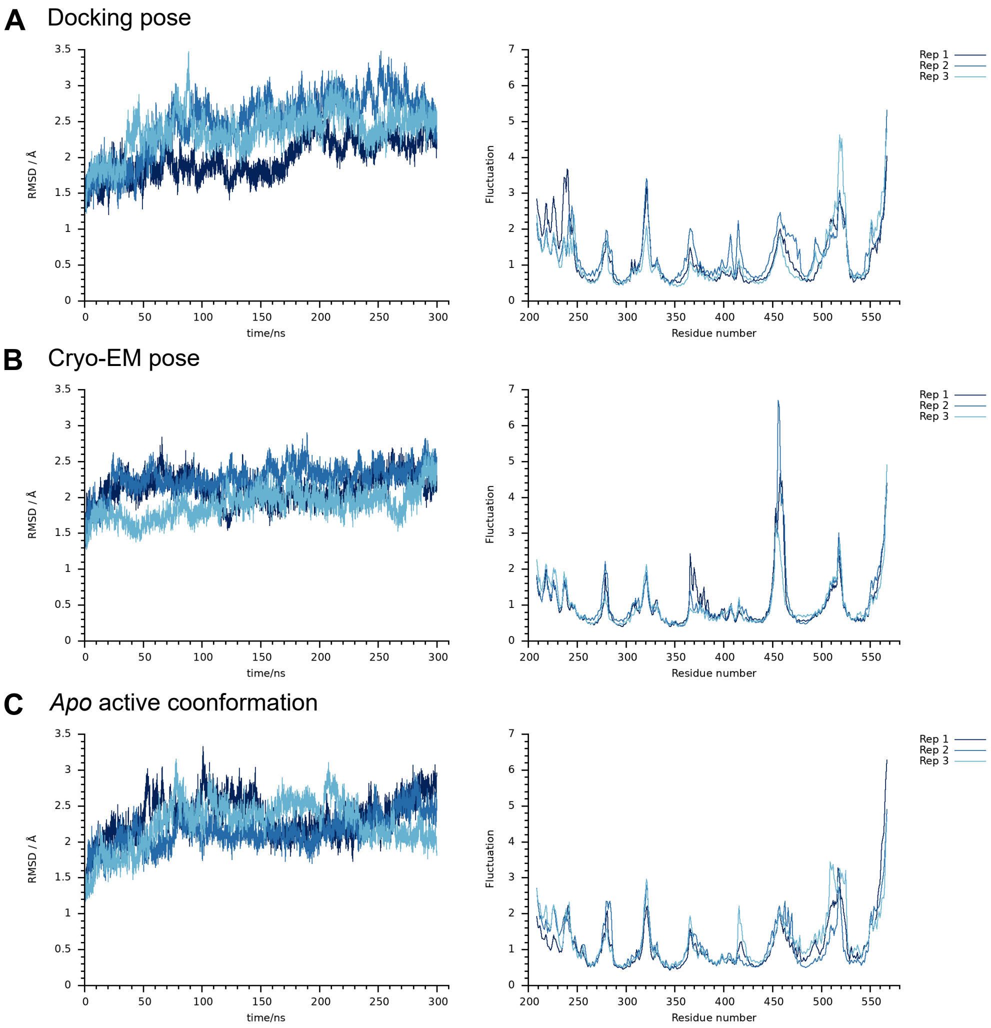

Supplementary Figure 13: RMSD and RMSF of FZD_7 during the MD simulations. Left: Full backbone RMSD of the entire protein and calculated in reference to the initial starting model. Right: RMSF of the full backbone of the protein plotted per residue. A: Starting from the complex of C407 and FZD_7 based on the docking calculations. B: Starting from the complex of C407 and FZD_7 based on the cryo-EM data. C: Starting from the receptor in an *apo* active conformation as used for the docking calculations. Data for the three independent replica are shown in different shades of blue.

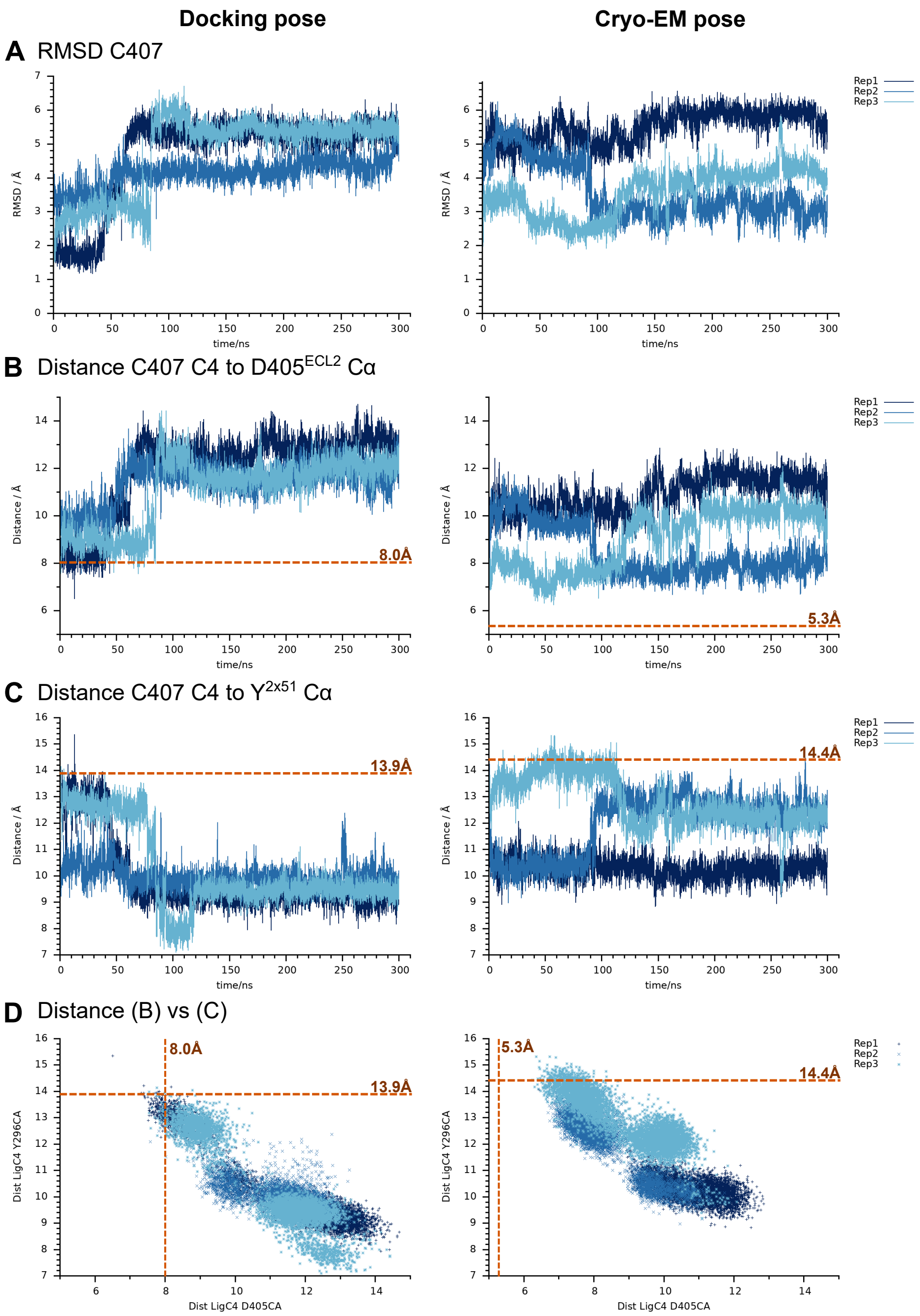

Supplementary Figure 14: Movement of C407 throughout the trajectories. Left: Starting from the complex of C407 and FZD_7 based on the docking calculations. Right: Starting from the complex of C407 and FZD_7 based on the cryo-EM data. A: RMSD of C407 heavy atoms in reference to the initial starting pose. B: Distance between C407 'C4' and the $D405^{ECL2}$ Cα atom. C: Distance between C407 'C4' and the $Y296^{2x51}$ Cα atom. D: Distances from B and C plotted against each other. See Supplementary Figure 16 for details on atoms used for the distance measurements. Orange lines and numbers indicate respective distances in the initial starting model. Data for the three independent replica are shown in different shades of blue.

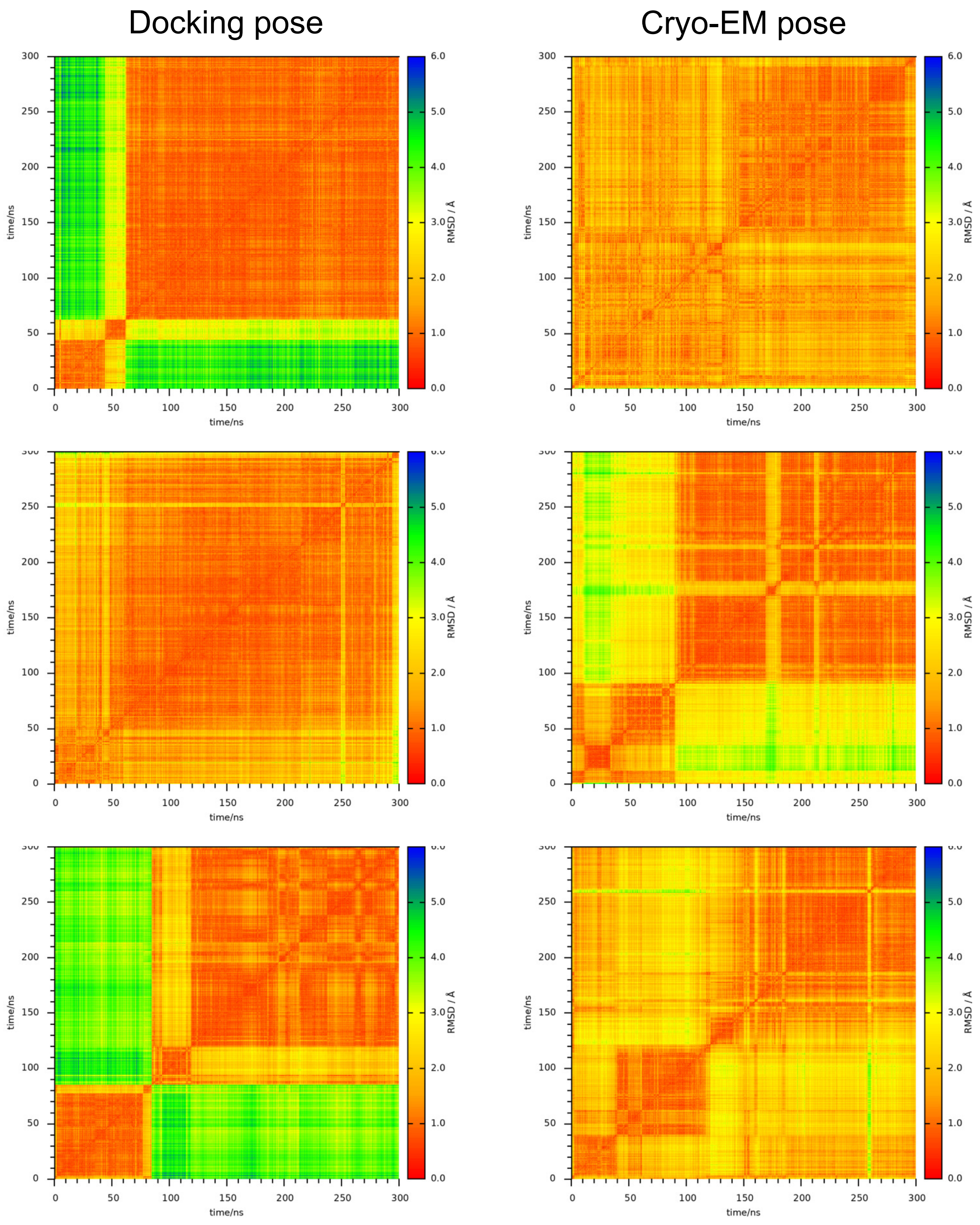

Supplementary Figure 15: 2D-RMS plots of C407 throughout the trajectories. Left: Starting from the complex of C407 and FZD_7 based on the docking calculations. Right: Starting from the complex of C407 and FZD_7 based on the cryo-EM data. Each plot is for one of the three independent replica comparing each frame of the trajectory with each frame of the same trajectory.

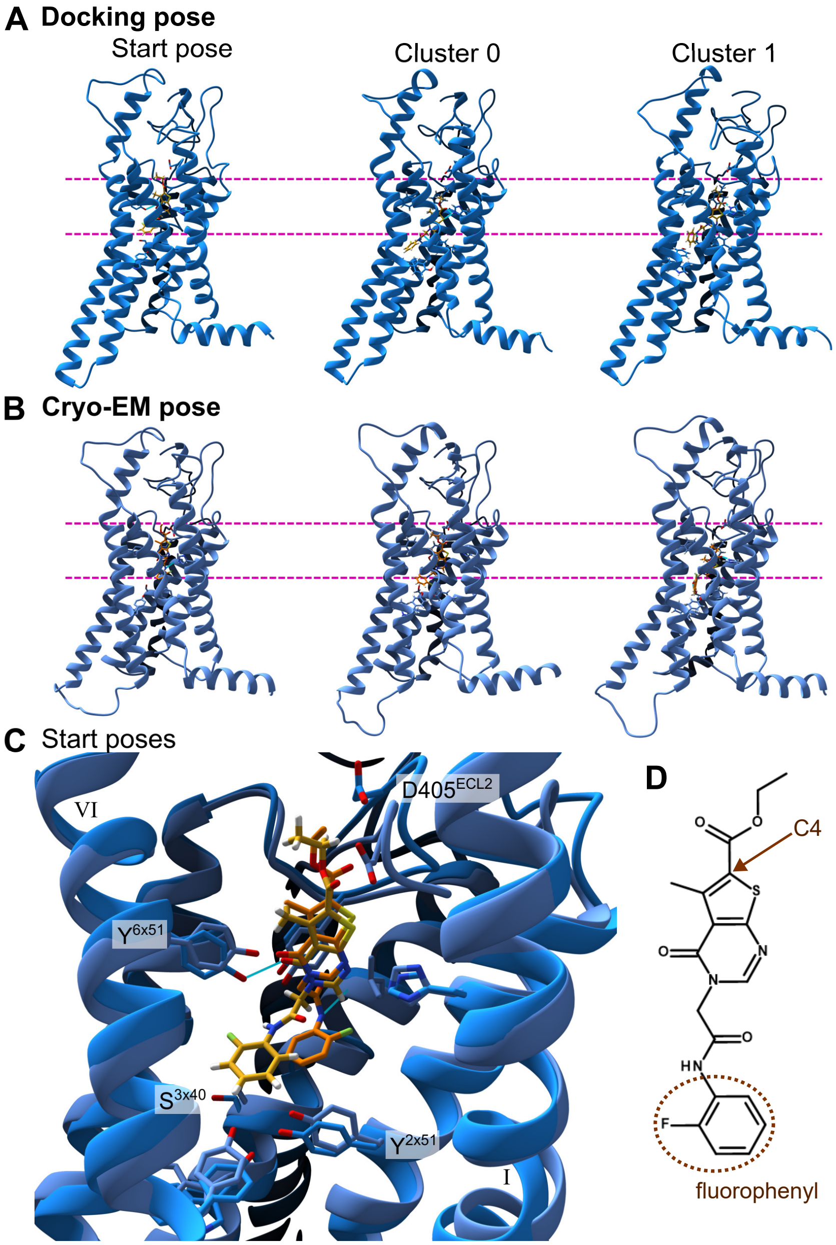

Supplementary Figure 16: Movement of C407 within the 7TMD throughout the MD simulations and definition of atoms for distance measurements. A: Side-view of the 7TMD of FZD_7 for MD simulations starting from the docking pose of C407. Left: Starting pose. Middle: Representative frame of the most frequent pose of C407 during the MD simulation (51 % of frames). Right: Representative frame of the second most frequent pose of C407 (35 % of frames). Pink dashed lines indicate top and bottom reach of C407 within the 7TMD for the initial model. B: Same as A but for the MD simulations starting from the cryo-EM pose. Cluster 0 comprises 56 % of frames and cluster 1 30 %. C: Closeup view of the starting poses of C407 in both MD simulation setups. Residues that were used for distance measurements are indicated with their respective numbering. D: Chemical structure of C407 with moieties and atoms used for distance measurements indicated. Corresponding distance measurements to C and D can be found in Figure 4 and Supplementary Figures 14 and 18.

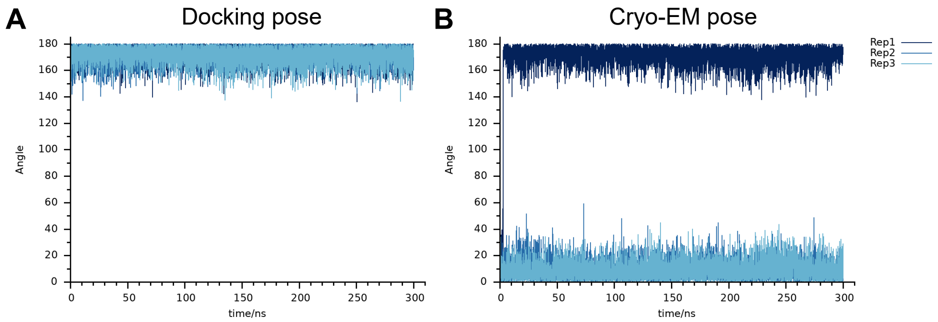

Supplementary Figure 17: Dihedral angle of the amide in C407. A: Starting from the complex of C407 and FZD_7 based on the docking calculations. B: Starting from the complex of C407 and FZD_7 based on the cryo-EM data. Data of the three independent replica are shown in different shades of blue.

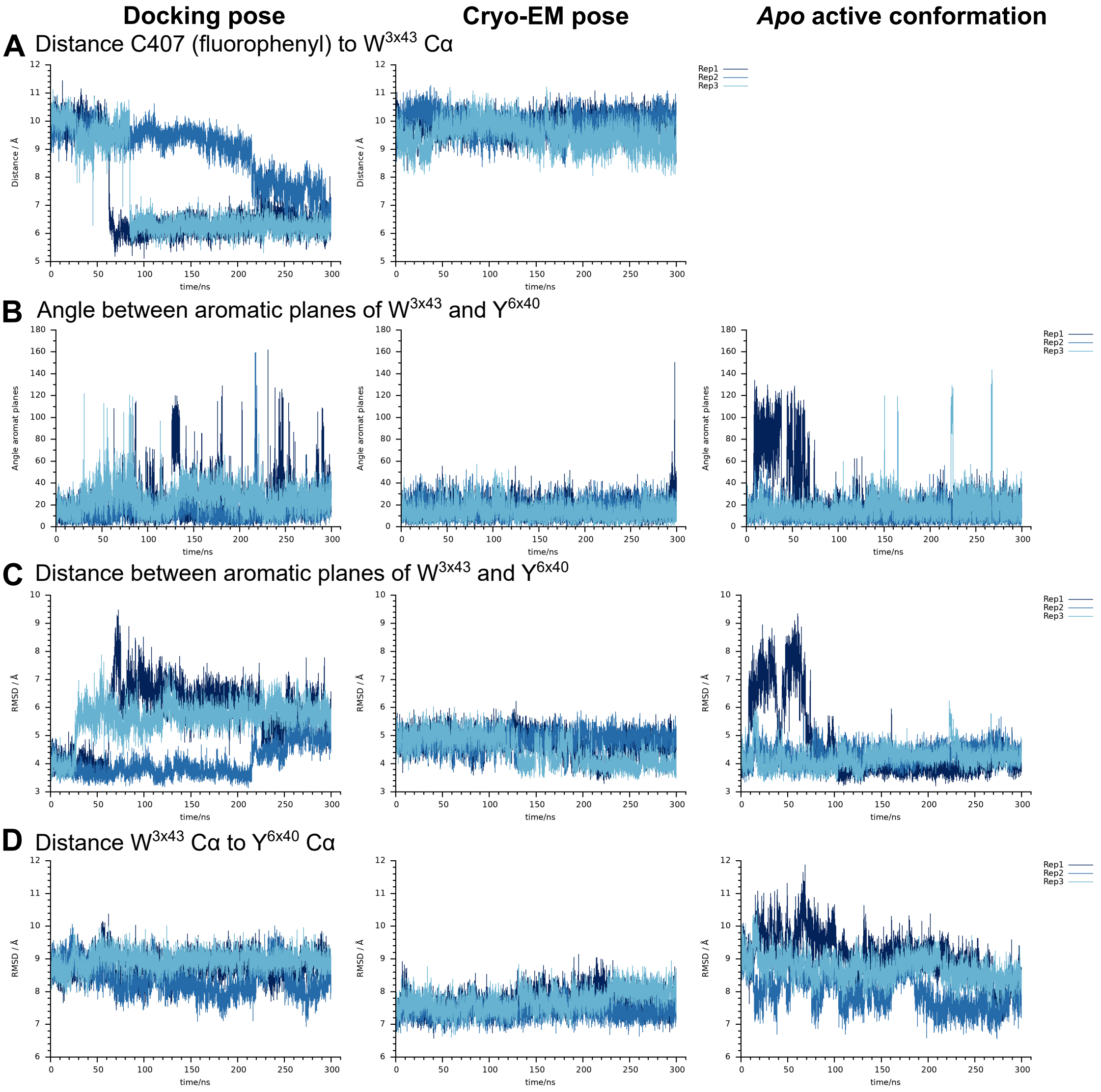

Supplementary Figure 18: Interplay between the movement of C407 and extended molecular residues W354^{3x43} and Y478^{6x40}. Left: Starting from the complex of C407 and FZD$_7$ based on the docking calculations. Middle: Starting from the complex of C407 and FZD$_7$ based on the cryo-EM data. Right: Starting from the receptor in an *apo* active conformation as used for the docking calculations. A: Distance between the center of mass of the C407 fluorophenyl group (see Supplementary Figure 16) and W354^{3x43} Cα. B: Angle between the vectors perpendicular to the aromatic planes of W354^{3x43} and Y478^{6x40}. C: Distance between the center of mass of the aromat rings of W354^{3x43} and Y478^{6x40}. D: Distance between Cα atoms of W354^{3x43} and Y478^{6x40}. Data for the three independent replica are shown in different shades of blue.

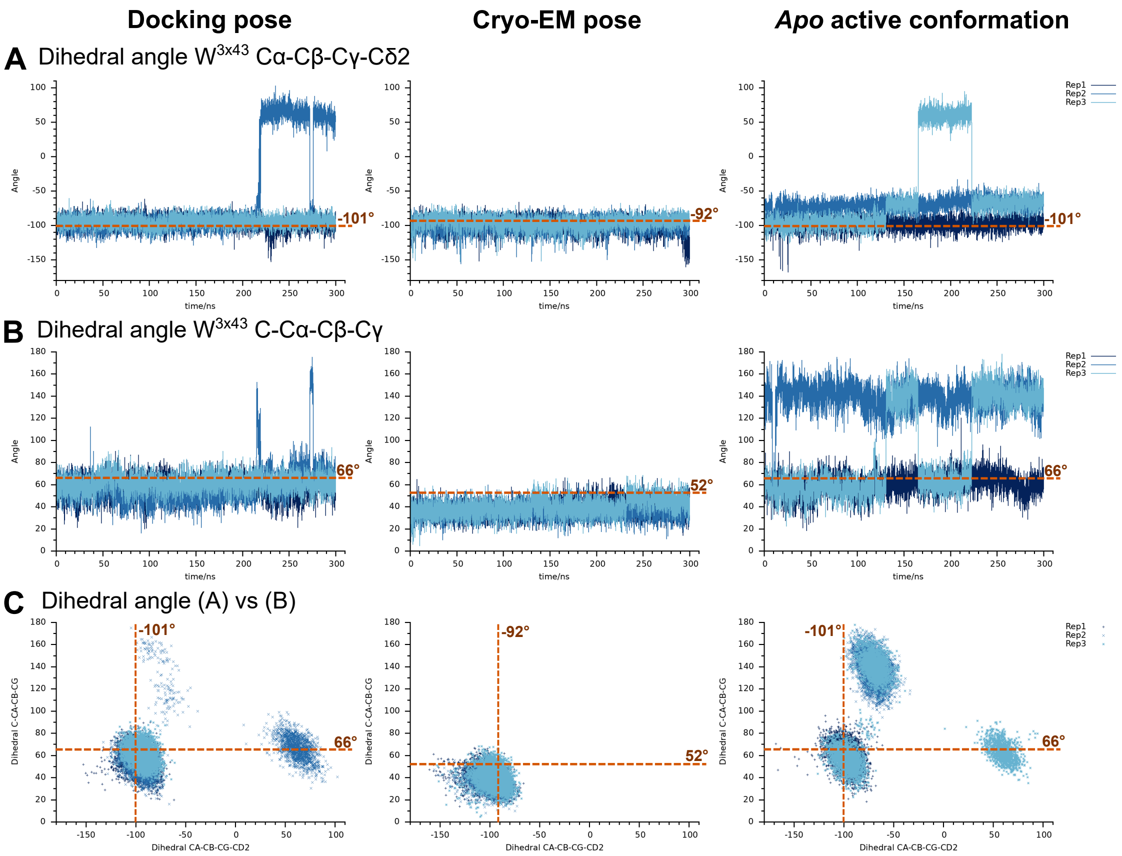

Supplementary Figure 19: Dihedral angels of the side chain of $W354^{3x43}$. Left: Starting from the complex of C407 and FZD_7 based on the docking calculations. Middle: Starting from the complex of C407 and FZD_7 based on the cryo-EM data. Right: Starting from the receptor in an *apo* active conformation as used for the docking calculations. A: Dihedral angle between Cα-Cβ-Cγ-Cδ2 atoms of $W354^{3x43}$. B: Dihedral angle between C-Cα-Cβ-Cγ atoms of $W354^{3x43}$. C: Dihedral angles from A and B plotted against each other. Orange lines and numbers indicate the respective dihedral angles in the initial starting model. Data of the three independent replica are shown in different shades of blue.

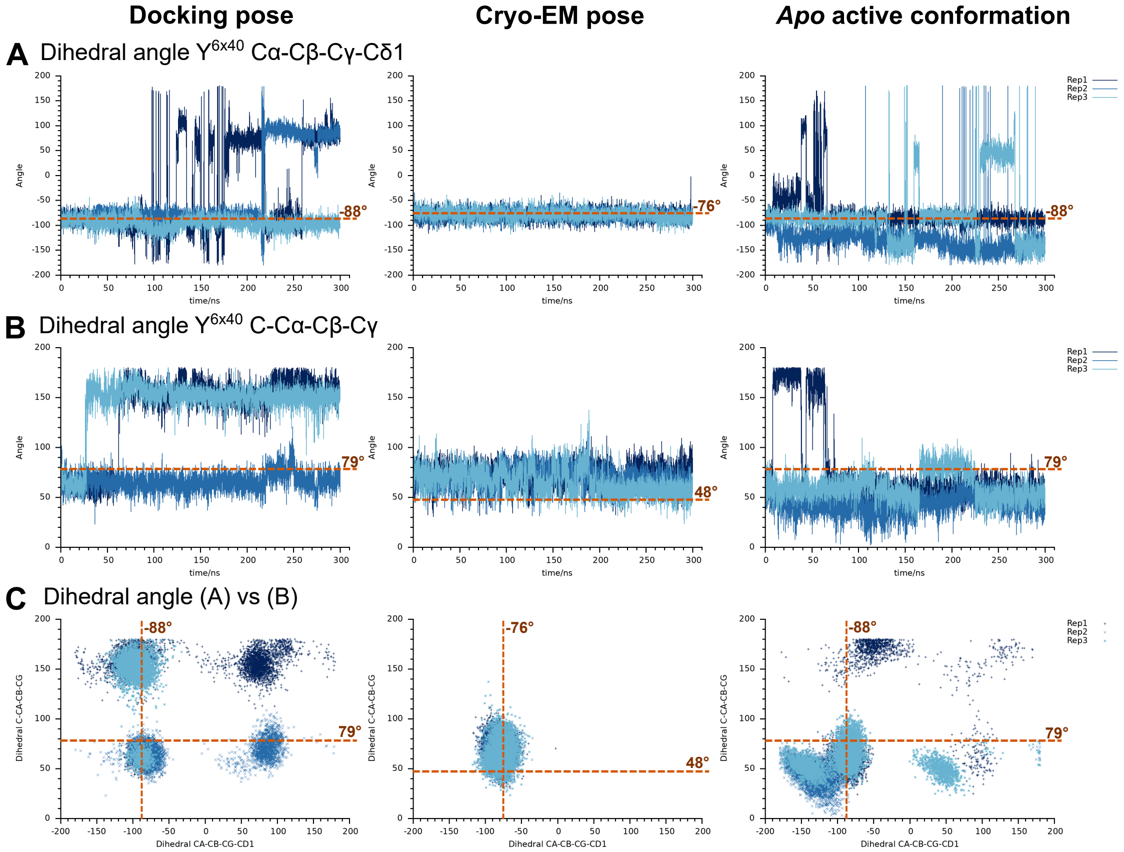

Supplementary Figure 20: Dihedral angels of the side chain of Y478^{6x40}. Left: Starting from the complex of C407 and FZD_7 based on the docking calculations. Middle: Starting from the complex of C407 and FZD_7 based on the cryo-EM data. Right: Starting from the receptor in an *apo* active conformation as used for the docking calculations. A: Dihedral angle between Cα-Cβ-Cγ-Cδ1 atoms of Y478^{6x40}. B: Dihedral angle between C-Cα-Cβ-Cγ atoms of Y478^{6x40}. C: Dihedral angles from A and B plotted against each other. Orange lines and numbers indicate the respective dihedral angles in the initial starting model. Data of the three independent replica are shown in different shades of blue.

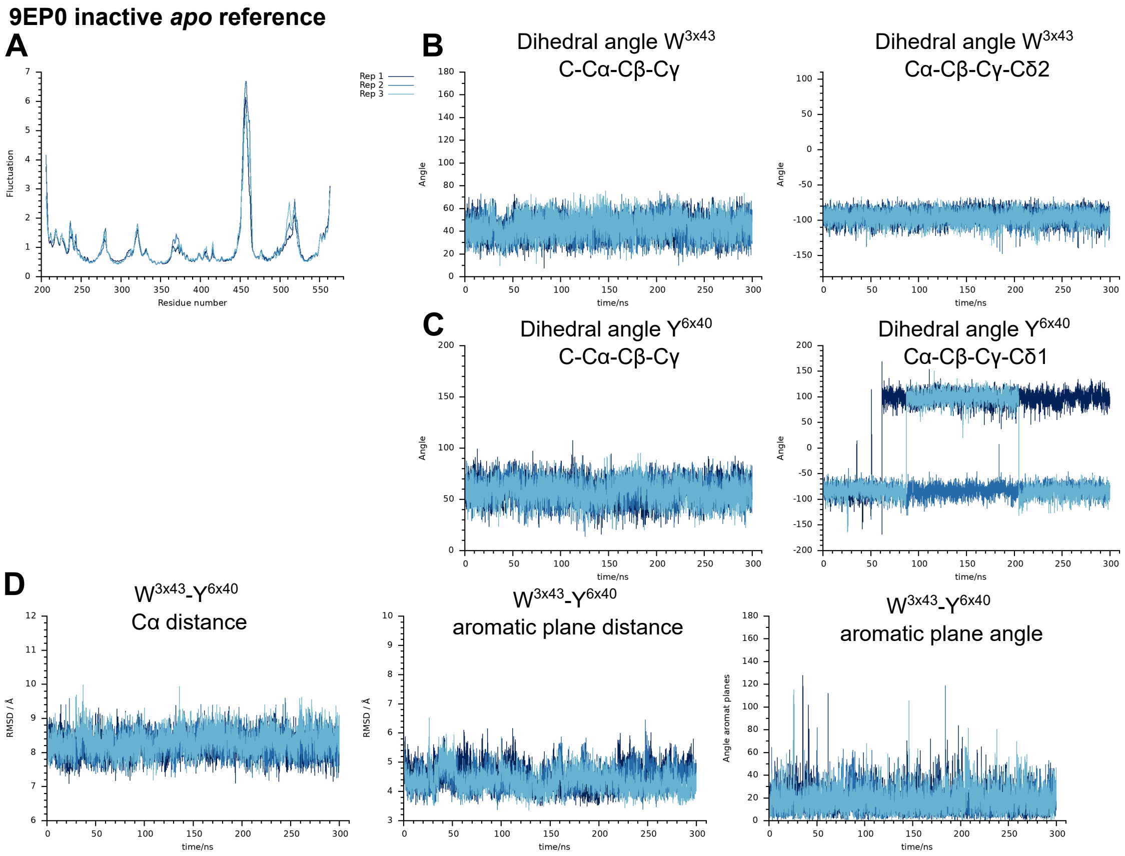

Supplementary Figure 21: Measurements of W354^{3x43} and Y478^{6x40} movement in the MD simulations of an *apo* inactive FZD$_7$ conformation. A: RMSF of the full backbone of the protein plotted per residue. B: Dihedral angle between C-Cα-Cβ-Cγ atoms and between Cα-Cβ-Cγ-Cδ2 atoms of W354^{3x43}. C: Dihedral angle between C-Cα-Cβ-Cγ atoms and between Cα-Cβ-Cγ-Cδ1 atoms of Y478^{6x40}. D: Distances between Cα atoms as well as distance and angles between the aromatic side chains of W354^{3x43} and Y478^{6x40}. The three different replica are plotted in different shades of blue. Data was taken from reference [1]. Of note, these MD simulations were run with a different force field than the MDs performed within this manuscript.

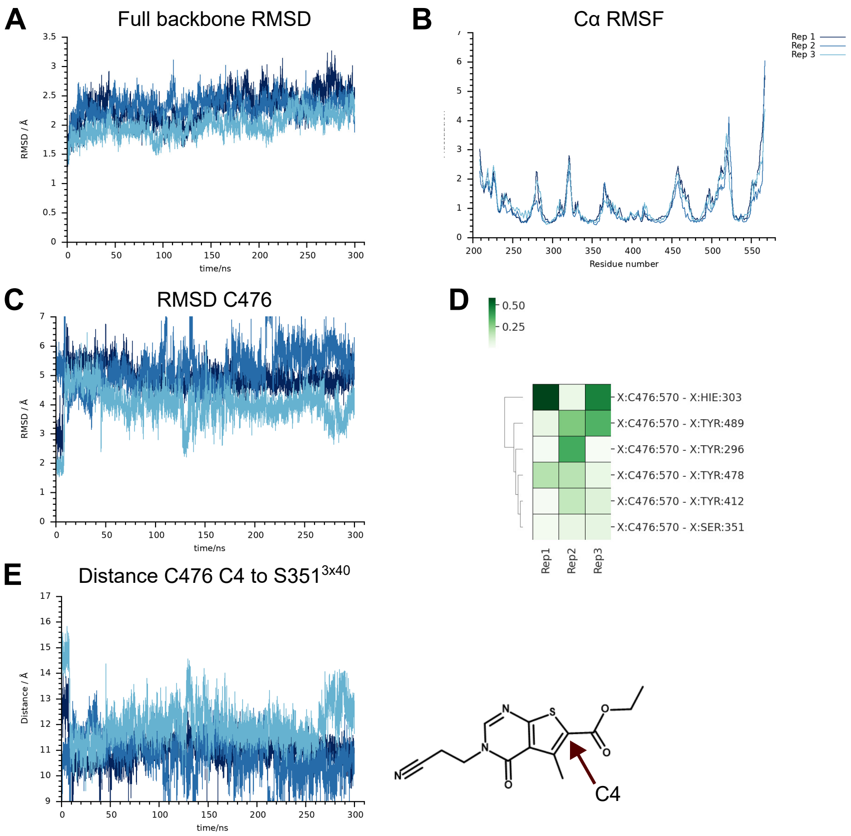

Supplementary Figure 22: Receptor and compound movements in the MD simulations with non-binder C476. A: Full backbone RMSD of FZD_7. B: RMSF of the Cα atoms over the course of the trajectories. C: RMSD of C476. D: Hydrogen bond interactions between C476 and residues of FZD_7 coloured by frequency. E: Distance between the C476 C4 atom and the Cα atom of C^{3x40}. The atom C4 is indicated in the chemical structure of C476 on the right. Time-courses for the three different replica are shown in different shades of blue.

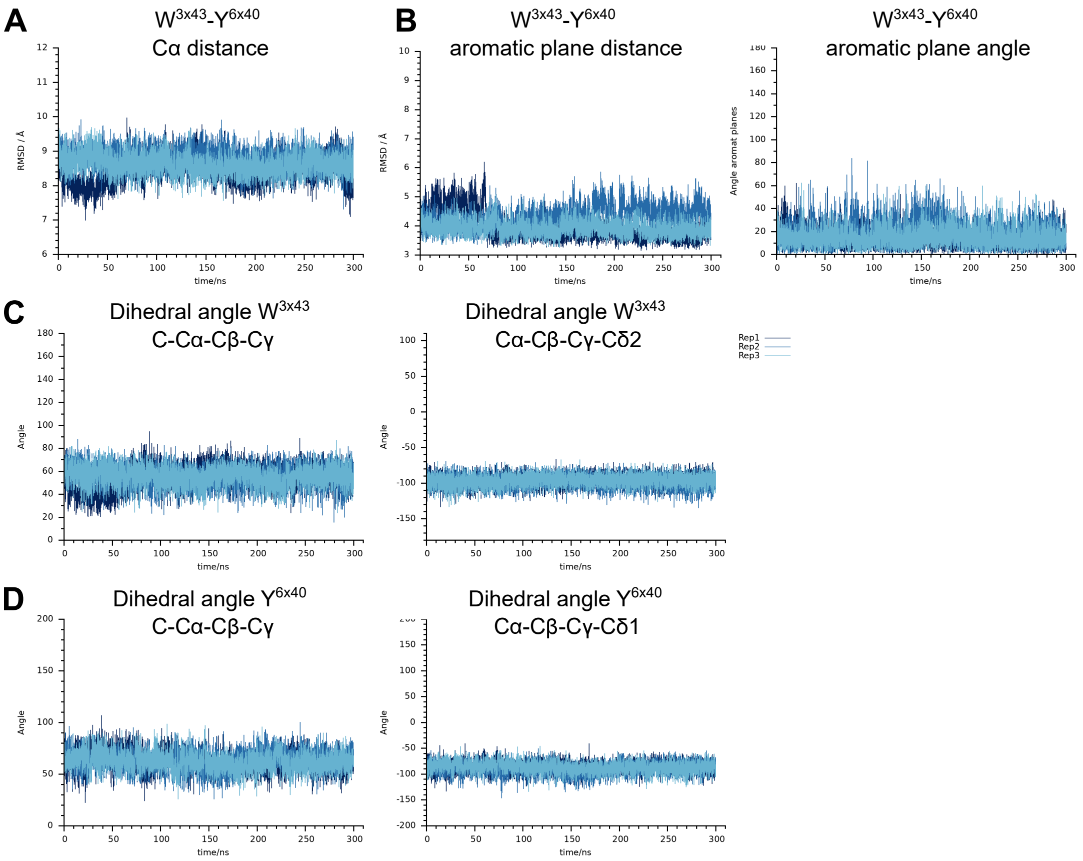

Supplementary Figure 23: Measurements of W354^{3x43} and Y478^{6x40} movements in the MD simulations starting from the docking pose of non-binder C476. A: Distances between Cα atoms of W354^{3x43} and Y478^{6x40}. B: Distance and angle between the aromatic side chains of W354^{3x43} and Y478^{6x40}. C: Dihedral angle between C-Cα-Cβ-Cγ atoms and between Cα-Cβ-Cγ-Cδ2 atoms of W354^{3x43}. D: Dihedral angle between C-Cα-Cβ-Cγ atoms and between Cα-Cβ-Cγ-Cδ1 atoms of Y478^{6x40}. Time-courses for the three independent replica are plotted in different shades of blue.

Supplementary Tables

Supplementary Table 1. Sequence alignment of residues within the potential binding site in the 7TMD of FZD_7. All residues within 5 Å distance of the docking poses of C407 and C45 and of the poses of C407 in the two first clusters of both MD simulations were extracted. Aligned residues are highlighted in a darker gray if they are the same as in FZD_7 or in a lighter gray if they have similar properties to the one in FZD_7. Sequence conservation as extracted from the GPCRdb is listed on the right. [2]

	Aligned FZD paralog sequences sorted by homology cluster										Sequence conservation (%)	
FZD_7	FZD_1	FZD_2	FZD_4	FZD_9	FZD_{10}	FZD_5	FZD_8	FZD_3	FZD_6	SMO	With SMO	Without SMO
$L292^{2x47}$	L358	L283	L258	L270	L266	L274	L316	Y241	Y237	V270	73	80
$Y296^{2x51}$	Y362	Y287	Y262	Y274	Y270	Y278	Y320	Y245	Y241	F274	91	100
$V299^{2x54}$	V365	V290	Y265	Y277	Y273	V281	V323	V248	V244	G277	64	70
$H303^{2x58}$	Y369	Y294	Y269	F281	Y277	F285	Y327	F252	Y248	W281	55	60
$L343^{3x32}$	L409	L334	M309	L320	L316	V322	V403	L293	L289	V321	64	70
$Y344^{3x33}$	Y410	Y335	Y310	Y321	Y317	Y323	Y404	Y294	Y290	Y322	100	100
$F346^{3x35}$	F412	F337	F312	F323	F319	F325	F406	F296	F292	A324	91	100
$G347^{3x36}$	S413	S338	G313	G324	G320	G326	G407	T297	T293	L325	55	60
$M348^{3x37}$	M414	M339	M314	M325	M321	M327	M408	M298	M294	M326	100	100
$S350^{3x39}$	S416	S341	S316	S327	S323	S329	S410	G300	G296	G328	73	80
$S351^{3x40}$	S417	S342	S317	S328	S324	S330	S411	S301	T297	V329	82	90

Continuation of Supplementary Table 1

	Aligned FZD paralog sequences sorted by homology cluster										Sequence conservation (%)	
FZD$_7$	FZD$_1$	FZD$_2$	FZD$_4$	FZD$_9$	FZD$_{10}$	FZD$_5$	FZD$_8$	FZD$_3$	FZD$_6$	SMO	With SMO	Without SMO
W354^{3x43}	W420	W345	W320	W331	W327	W333	W414	W304	W300	F332	91	100
D405^{ECL2}	D471	D396	D371	D382	D378	D384	D465	D355	D351	D384	100	100
L407^{ECL2}	L473	L398	L373	L384	L380	V386	V467	I357	I353	V386	55	60
S408^{ECL2}	S474	S399	T374	T385	T381	A387	A468	S358	S354	S387	55	50
V410^{ECL2}	V476	V401	L376	L387	V383	I389	I470	V360	V356	I389	55	60
C411^{45x50}	C477	C402	C377	C388	C384	C390	C471	C361	C357	C390	100	100
Y412^{45x51}	F478	F403	Y378	Y389	Y385	Y391	Y472	F362	F358	F391	55	60
V413^{45x52}	V479	V404	V379	V390	V386	V392	V473	V363	V359	V392	100	100
L415^{ECL2}	L481	L406	N381	S392	S388	N394	N475	L365	L361	Y394	45	50
V425^{5x47}	V491	V416	V391	V402	V398	V404	V485	V375	V371	V404	100	100
L429^{5x51}	L495	L420	L395	L406	L402	L408	L489	L379	L375	I408	91	100
Y432^{5x54}	Y498	Y423	Y398	Y409	Y405	Y411	Y492	Y382	C378	V411	82	90
L433^{5x55}	L499	L424	L399	L410	L406	L412	L493	V383	V379	L412	82	80
G436^{5x58}	G502	G427	G402	G413	G409	G415	G496	G386	G382	G415	100	100
L477^{6x39}	L543	L468	L443	L454	L450	L456	L539	L427	L423	L458	100	100
Y478^{6x40}	Y544	Y469	Y444	Y455	Y451	Y457	Y540	Y428	Y424	A459	91	100

Continuation of Supplementary Table 1

	Aligned FZD paralog sequences sorted by homology cluster										Sequence conservation (%)	
FZD_7	FZD_1	FZD_2	FZD_4	FZD_9	FZD_{10}	FZD_5	FZD_8	FZD_3	FZD_6	SMO	With SMO	Without SMO
T479^{6x41}	T545	T470	T445	T456	T452	T458	T541	L429	L425	F460	73	80
P481^{6x43}	P547	P472	P447	P458	P454	P460	P543	P431	P427	F462	91	100
A482^{6x44}	A548	A473	A448	A459	A455	A461	A544	L432	L428	V463	73	80
V485^{6x47}	V551	V476	V451	V462	V458	V464	V547	V435	L431	T466	82	90
L486^{6x48}	I552	I477	I452	I463	I459	V465	V548	I436	L432	F467	55	60
Y489^{6x51}	Y555	Y480	Y455	Y466	Y462	Y468	L551	Y439	Y435	H470	82	90
E492^{6x54}	E558	E483	E458	E469	E465	E471	E554	E442	E438	D473	91	100
F530^{7x38}	F603	Y521	E477	F510	F504	L503	F586	F480	F476	E518	64	70
K533^{7x41}	K606	K524	K480	K513	K507	K506	K589	K483	K479	N521	91	100
Y534^{7x42}	Y607	Y525	I481	I514	I508	Y507	Y590	Y484	Y480	L522	64	70
M536^{7x44}	M609	M527	M483	M516	M510	M509	M592	M486	M482	A524	91	100
T537^{7x45}	T610	T528	S484	S517	L511	C510	C593	A487	T483	M525	36	40
M538^{7x46}	L611	L529	L485	L518	L512	L511	L594	L488	L484	F526	82	90
I539^{7x47}	I612	I530	L486	V519	V513	V512	V595	I489	I485	G527	45	50
V540^{7x48}	V613	V531	V487	V520	V514	V513	V596	V490	V486	T528	91	100
G541^{7x49}	G614	G532	G488	G521	G515	G514	G597	G491	G487	G529	100	100

Continuation of Supplementary Table 1

	Aligned FZD paralog sequences sorted by homology cluster										Sequence conservation (%)	
FZD_7	FZD_1	FZD_2	FZD_4	FZD_9	FZD_{10}	FZD_5	FZD_8	FZD_3	FZD_6	SMO	With SMO	Without SMO

Supplementary Table 2. Cryo-EM data collection, refinement, and validation statistics.

	inactive FZD_7 EMDB-53969/ PDB: 9RHG
**Data collection and processing**	
Magnification	165,000
Voltage (kV)	300
Defocus range (µM)	-0.4 -1.8
Pixel size (Å)	0.507
Symmetry imposed	C1
Initial particle images (no.)	19,899
Map resolution	
FSC threshold	0.143
Map local resolution range (Å)	2.13-3.43
**Refinement**	
Initial models used	9EPO
Model resolution (Å) (FSC=0.143)	1.9
Map sharpening B factor (Å^2)	-30
Model composition	
Non-hydrogen atoms	5767
Number of protein residues/atoms	694
Number of ligands/ligand atoms	5
Average B factor (Å^2)	
Protein	51.37
Ligands	34.83
R.m.s deviations	
Bond lengths (Å)	0.003
Bond angles (°)	0.669

Continuation of Supplementary Table 2.

	inactive FZD_7 EMDB-53969/ PDB: 9RHG
Validation	
Molprobity score	1.35
Clashscore	3.91
Poor rotamers (%)	1.69
Ramachandran plot	
Favored (%)	98.25
Allowed (%)	1.75
Disallowed (%)	0

EMD; electron microscopy bank, PDB; protein data bank, RMSD; root mean square deviation.

Supplementary Table 3. System setup parameters for all included MD simulations.

	apo active	Docking pose	Cryo-EM pose	Non-binder C476
Box dimensions	85x85x120	85x85x120	85x85x120	85x85x120
Total no. of atoms	71288	71352	70425	71303
No. of water molecules	15198	15205	14852	15192
Salt concentration [mM]	150	150	150	150
Lipid composition				
Lipid type	POPC	POPC	POPC	POPC
No. of lipids (Top)	75	75	75	75
No. of lipids (Bottom)	73	73	74	73
Simulation length				
Equilibration NVT [ns]	0.25	0.25	0.25	0.25
Equilibration NPT [ns]	16.75	16.75	16.75	16.75
Production runs [ns]	300	300	300	300
Number of replica	3	3	3	3

Supplementary Table 4. Root-mean-square fluctuation (RMSF) of Cα atoms and the side-chain heavy atoms of W354^{3x43} and Y478^{6x40}. Values were calculated for each replica separately (p1-p3) over the entire trajectory of each MD simulation. MD simulations with an active FZD_7 conformation are highlighted in grey.

		W3x43		Y6x40	
		Cα	side-chain	Cα	Side-chain
apo active	p1	0.476	0.659	1.009	2.959
	p2	0.554	0.918	0.753	1.354
	p3	0.624	2.189	1.439	2.492
Docking pose	p1	0.531	0.719	0.668	2.479
	p2	0.819	2.174	1.537	2.191
	p3	0.409	0.559	0.587	1.839
Cryo-EM pose	p1	0.453	0.615	0.535	0.869
	p2	0.418	0.518	0.494	0.819
	p3	0.388	0.645	0.708	1.384
apo inactive[a]	p1	0.435	0.649	0.708	1.295
	p2	0.469	0.667	0.764	1.012
	p3	0.464	0.684	0.646	1.272
Non-binder C476	p1	0.584	0.771	0.639	1.071
	p2	0.450	0.606	0.502	0.762
	p3	0.493	0.592	0.565	0.844

[a] Data was taken from reference [1]. Of note, these MD simulations were run with a different force field.

Supplementary Table 5. Vendor and compound identifiers for all tested compounds as well as compound purities as provided by vendor quality control data. The hit molecules identified in pharmacological assays are highlighted in grey.

ID	ZINC ID	MolPort ID	Vendor ID	Vendor	purity
C11	ZINC000006069641		sc-210120B	SantaCruz	≥95%
C12	ZINC000065408474	MolPort-047-489-699	53943877	ChemBridge	100%
C13	ZINC000072126504	MolPort-047-494-752	16054903	ChemBridge	93%
C14	ZINC000072419084	MolPort-047-495-109	34193291	ChemBridge	97%
C15	ZINC000077321841	MolPort-047-497-053	23318776	ChemBridge	100%
C16	ZINC000163762760		Z1515110521	Enamine	97%
C17	ZINC000328654785		Z1757898504	Enamine	95%
C21	ZINC000006702107	MolPort-000-832-620	9019362	ChemBridge	≥85%
C22	ZINC000097604206	MolPort-047-503-159	57305870	ChemBridge	94%
C31	ZINC000000812166		Z46168873	Enamine	100%
C32	ZINC000067805280	MolPort-019-816-663	70332011	ChemBridge	100%
C33	ZINC000067934644	MolPort-019-821-985	87464791	ChemBridge	95%
C34	ZINC000072898705		Z1301343910	Enamine	96%
C35	ZINC000095421423		Z1439375842	Enamine	98%
C36	ZINC000151702594		Z1458340850	Enamine	100%
C37	ZINC000178964316		Z2242915358	Enamine	100%
C38	ZINC000244787808	MolPort-039-034-783	16750652	ChemBridge	100%
C41	ZINC000000142135	MolPort-000-662-104	6541524	ChemBridge	≥85%
C42	ZINC000000618889	MolPort-001-580-765	STK149302	Vitas-M	≥85%
C43	ZINC000005685988	MolPort-001-558-552	7657461	ChemBridge	≥85%
C44	ZINC000013127563	MolPort-002-597-530	STL221649	Vitas-M	≥85%
C45	ZINC000014128943		Z88129578	Enamine	89%
C401			CBK448805	CBCS library	98%

Continuation of Supplementary Table 5.

ID	ZINC ID	MolPort ID	Vendor ID	Vendor	purity
C402			CBK413891	CBCS library	99%
C403			CBK249809	CBCS library	94%
C404			CBK071900	CBCS library	99%
C405			CBK268514	CBCS library	94%
C406			CBK072830	CBCS library	93%
C407[a]			CBK434718	CBCS library	98%
C407			Z25040997	Enamine	100%
C408			CBK304562	CBCS library	85%
C409			CBK449777	CBCS library	98%
C410			CBK297102	CBCS library	55%
C411			CBK071910	CBCS library	99%
C471			Z202575498	Enamine	94%
C472			Z94735417	Enamine	98%
C473			Z87739603	Enamine	94%
C474			Z26743350	Enamine	94%
C475			Z25040877	Enamine	94%
C476			Z54342035	Enamine	100%
C477			Z94736991	Enamine	97%
C478			Z107063344	Enamine	93%

[a] C407 from this source was only used for initial binding experiments. All other experiments were conducted with C407 from Enamine.

Supplementary Table 6. Compound SMILES for all tested compounds.

ID	SMILES
C11	Cc1cc(=O)oc2cc(OC(=O)c3ccc(N=C(N)N)cc3)ccc12
C12	O=C(N(Cc1ccc(F)cc1)Cc1ccccn1)C12CNCC1CNC2
C13	CN(Cc1nc(-c2ccccc2)no1)C(=O)[C@H]1CC2(CCNCC2)CN1
C14	Cc1ccc(OCCNC[C@]2(O)CCCNC2)cc1C
C15	O[C@]1(CNCc2ccc(-n3cccn3)cc2)CNCCOC1
C16	NCC[C@H]1CCCN(Cc2ccc(F)c(C(F)(F)F)c2)C1
C17	CN1CC[C@@H](CNCc2cccc3nccn23)[C@@H]1c1cccnc1
C21	Brc1ccc(OCc2ccccc2)c(CNCc2cccnc2)c1
C22	NCC[C@H]1CN(Cc2ccc(Oc3ccccc3)cc2)CCO1
C31	O=C(CN1CCCC1)Nc1ccc(Br)cc1
C32	Cc1ccc(O)c(CN2CCC([C@]3(c4cccnc4)NC(=O)NC3=O)CC2)c1
C33	COc1ccc(Oc2nc(C)cnc2C)cc1CN1CCCC1
C34	Cc1ccc(NC(=O)CN2CCC3(CCOCC3)C2)cc1
C35	O=C(Nc1ccc(CN[C@@H]2CCc3ccccc32)cc1)c1cccnc1
C36	O=C(CNC[C@H]1CCCO1)Nc1ccc(-c2csnn2)cc1
C37	CNC[C@@H]1CCN(C(=O)[C@H]2COc3ccc(Cl)cc3C2)C1
C38	O=C(CN1CCC2(CC1)c1ccccc1C[C@H]2O)Nc1ccc(F)cc1
C41	Cc1ccc(N2C(=O)C(Cl)=C(Nc3cccc(O)c3)C2=O)c(C)c1
C42	CCOc1cccc(N2C(=O)C[C@H](n3cnc4ccccc43)C2=O)c1
C43	COC(=O)c1ccc(NC(=O)CSc2nc(CC(C)C)n[nH]2)cc1
C44	COc1ccc([C@@H]2C[C@H](c3ccc(C)cc3)Nc3nc(N)nn32)cc1
C45	Cc1c(-c2ccccc2)sc2ncn(CC(=O)NCc3ccc4c(c3)OCO4)c(=O)c12
C401	s1c2ncn(c(=O)c2cc1c1ccccc1)CC(=O)OCCC
C402	s1c2ncn(c(=O)c2c2c1CCCC2)CC(=O)NCc1ccccc1
C403	c12c(ncn(c1=O)CC(=O)NCCOc1cc(Cl)ccc1)sc(c2C)C

Continuation of Supplementary Table 6.

ID	Smiles
C404	c12c(ncn(c1=O)CC(=O)NCc1ncccc1)sc(c2)C
C405	c12c(sc(c1C)C(=O)N)ncn(c2=O)CC(=O)NCCc1ccccc1
C406	n1(c(=O)c2c(nc1C)cccc2)CC(=O)NCc1ccccc1
C407	s1c2ncn(c(=O)c2c(c1C(=O)OCC)C)CC(=O)Nc1c(F)cccc1
C408	c12c(ncn(c1=O)CC(=O)NC1c3c(OCC1)cccc3)sc(c2C)C
C409	s1c2ncn(c(=O)c2c(c1C(=O)N)C)CC(=O)Nc1c(cccc1)CC
C410	c12c(ncn(c1=O)CC(=O)c1cc3c(OCO3)cc1)sc(c2)c1ccccc1
C411	c12c(sc(c1C)C(=O)Nc1c(OC)cccc1)ncn(c2=O)CC(=O)NCCC
C471	Cc1c(-c2ccccc2)sc(N=CN2CC(Nc3c(C(OC)=O)scc3)=O)c1C2=O
C472	CCOC(c1c(C)c(C(N(CC(Nc2cc(OC)ccc2)=O)C(C)=N2)=O)c2s1)=O
C473	O=C(CN(C=Nc1c2c(CCC3)c3s1)C2=O)Nc(c(F)ccc1)c1F
C474	Cc1c(C)sc(N=CN2CC(Nc(cc3)cc(Cl)c3OC)=O)c1C2=O
C475	CCOC(c1c(C)c(C(N(CC(Nc(cc2)cc3c2OCO3)=O)C=N2)=O)c2s1)=O
C476	CCOC(c1c(C)c(C(N(CCC#N)C=N2)=O)c2s1)=O
C477	CCCC(C)NC(CN(C(C)=Nc1c2c(C)c(C(OCC)=O)s1)C2=O)=O
C478	Cc1c(C(O)=O)sc(N=CN2CC(NCc(cc3)ccc3F)=O)c1C2=O

Supplementary References

1. Bous, J. *et al.* Structural basis of frizzled 7 activation and allosteric regulation. *Nature Communications* **15,** 7422 (2024).

2. Isberg, V. *et al.* GPCRDB: an information system for G protein-coupled receptors. *Nucleic Acids Research* **42,** D422–D425 (2013).
