# Supplementary material for: In silico docking yields small molecule negative allosteric modulators targeting the core of Frizzled 7
Source: Nat Commun. 2025 Dec 14;16:11138. doi: 10.1038/s41467-025-67147-z (PMC12705740; doi:10.1038/s41467-025-67147-z)
Supplement: Supplementary file 2 — Description of Additional Supplementary Files [file 41467_2025_67147_MOESM2_ESM.pdf]

Description of Additional Supplementary Files

File name: Supplementary Data 1

Description: Docking poses. Docking poses of C45 and C407 as well as C471-C478 in complex with the FZD_7 receptor structure as used for the respective docking calculations (.pdb files).

File name: Supplementary Data 2

Description: FZD_7 density map. Density map of the detergent solubilized FZD_7 antiparallel dimer in complex with C407.

File name: Supplementary Data 3

Description: FZD model. Model of the FZD_7 antiparallel dimer in complex with C407.

File name: Supplementary Data 4

Description: MD clusters. Centroid structures of C407 in complex with FZD_7 from the MD simulations after clustering based on C407 poses (.pdb files)

File name: Supplementary Data 5

Description: MD input. MD parameter files for minimisation, equilibration and production as well as initial and final configurations of each MD system (.pdb files).

File name: Supplementary Data 6

Description: MD simulation checklist. Molecular Dynamics simulation checklist to help guide readers to information about reproducibility of the simulations.
