# Supplementary material for: In silico docking yields small molecule negative allosteric modulators targeting the core of Frizzled 7
Source: Nat Commun. 2025 Dec 14;16:11138. doi: 10.1038/s41467-025-67147-z (PMC12705740; doi:10.1038/s41467-025-67147-z)
Supplement: Supplementary file 8 — Supplementary Data 6 [file 41467_2025_67147_MOESM8_ESM.pdf]

**Reliability and reproducibility checklist for molecular dynamics simulations**<br>***All boxes must be marked YES by acceptance unless "Response not needed if No".**	**Yes**	**No**	**Response**<br>**(Please state where this information can be found in the text)**
**1. Convergence of simulations and analysis**			
1a. Is an evaluation presented in the text to show that the property being measured has equilibrated in the simulations (*e.g.* time-course analysis)?	☒	☐	Time-courses for measurements can be found in Figure 4 and in the Supplementary Information
1b. Then, is it described in the text how simulations are split into equilibration and production runs and how much data were analyzed from production runs?	☒	☐	The simulation protocol is described in the methods section
1c. Are there at least 3 simulations per simulation condition with statistical analysis?	☒	☐	
1d. Is evidence provided in the text that the simulation results presented are independent of initial configuration?	☒	☐	The last equilibration step was restarted with new random velocities for each replica. Simulations starting from the docking pose and a structural pose lead to overall similar results.
**2. Connection to experiments**			
2a. Are calculations provided that can connect to experiments (*e.g.* loss or gain in function from mutagenesis, binding assays, NMR chemical shifts, J-couplings, SAXS curves, interaction distances or FRET distances, structure factors, diffusion coefficients, bulk modulus and other mechanical properties, *etc*.)?	☒	☐	Connected experimental data obtained by mutagenesis of a FZD biosensor can be found in Fig. 5, Fig. S7 and explained in the results section.
**3. Method choice**			
3a. Do simulations contain membranes, membrane proteins, intrinsically disordered proteins, glycans, nucleic acids, polymers, or cryptic ligand binding?	☒	☐	Membrane protein embedded in a membrane bilayer
3b. Is it described in the text whether the accuracy of the chosen model(s) is sufficient to address the question(s) under investigation (e.g. all-atom vs. coarse-grained models, fixed charge vs. polarizable force fields, implicit vs. explicit solvent or membrane, force field and water model, etc.)?	☒	☐	Methods section:<br>"The aim of the MD simulations was to assess the stability of the binding pose and interactions of C407 within its binding site in the 7TMD core of FZD_7. For this purpose, all-atom simulations with explicit solvent are suitable to describe the desired observables.<br>All MD simulations were run using GROMACS 2024

			and AMBER force fields (protein: FF19SB; lipids: Lipid21; water: TIP3P), which are suitable to describe membrane protein systems and interactions with small molecules.”
3c. Is the timescale of the event(s) under investigation beyond the brute-force MD simulation timescale in this study that enhanced sampling methods are needed?	☐	☒	
If **YES**, are the parameters and convergence criteria for the enhanced sampling method clearly stated?	☐	☐	
If **NO,** is the evidence provided in the text?	☒	☐	Methods section: “This time scale is sufficient to investigate the stability of a bound compound within the binding site.”
**4. Code and reproducibility**			
4a. Is a table provided describing the system setup that includes simulation box dimensions, total number of atoms, total number of water molecules, salt concentration, lipid composition (number of molecules and type)?	☒	☐	Supplementary Table S3
4b. Is it described in the text what simulation and analysis software and which versions are used?	☒	☐	Methods section
4c. Are other parameters for the system setup described in the text, such as protonation state, type of structural restraints if applied, nonbonded cutoff, thermostat and barostat, etc.?	☒	☐	Methods section
4d. Are initial coordinate and simulation input files and a coordinate file of the final output provided as supplementary files or in a public repository?	☒	☐	Simulation input files and trajectories are deposited in the GPCRmd (https://gpcrmd.org/)
4e. Is there custom code or custom force field parameters?	☒	☒	Response not needed if **No**
If **YES**, are they provided as supplementary files or in a public repository?	☒	☐	Custom force field parameters for C407 and C476 are deposited in the GPCRmd (https://gpcrmd.org/)
