# Supplementary material for: In silico docking yields small molecule negative allosteric modulators targeting the core of Frizzled 7
Source: Nat Commun. 2025 Dec 14;16:11138. doi: 10.1038/s41467-025-67147-z (PMC12705740; doi:10.1038/s41467-025-67147-z)
Supplement: Supplementary file 9 — Reporting Summary [file 41467_2025_67147_MOESM9_ESM.pdf]

nature portfolio

Corresponding author(s): Gunnar Schulte

Last updated by author(s): Nov 10, 2025

# Reporting Summary

Nature Portfolio wishes to improve the reproducibility of the work that we publish. This form provides structure for consistency and transparency in reporting. For further information on Nature Portfolio policies, see our Editorial Policies and the Editorial Policy Checklist.

## Statistics

For all statistical analyses, confirm that the following items are present in the figure legend, table legend, main text, or Methods section.

| n/a | Confirmed | |
|---|---|---|
| ☐ | ☒ | The exact sample size (*n*) for each experimental group/condition, given as a discrete number and unit of measurement |
| ☐ | ☒ | A statement on whether measurements were taken from distinct samples or whether the same sample was measured repeatedly |
| ☐ | ☒ | The statistical test(s) used AND whether they are one- or two-sided<br>*Only common tests should be described solely by name; describe more complex techniques in the Methods section.* |
| ☒ | ☐ | A description of all covariates tested |
| ☐ | ☒ | A description of any assumptions or corrections, such as tests of normality and adjustment for multiple comparisons |
| ☐ | ☒ | A full description of the statistical parameters including central tendency (e.g. means) or other basic estimates (e.g. regression coefficient) AND variation (e.g. standard deviation) or associated estimates of uncertainty (e.g. confidence intervals) |
| ☐ | ☒ | For null hypothesis testing, the test statistic (e.g. *F*, *t*, *r*) with confidence intervals, effect sizes, degrees of freedom and *P* value noted<br>*Give P values as exact values whenever suitable.* |
| ☒ | ☐ | For Bayesian analysis, information on the choice of priors and Markov chain Monte Carlo settings |
| ☒ | ☐ | For hierarchical and complex designs, identification of the appropriate level for tests and full reporting of outcomes |
| ☒ | ☐ | Estimates of effect sizes (e.g. Cohen's *d*, Pearson's *r*), indicating how they were calculated |

*Our web collection on statistics for biologists contains articles on many of the points above.*

## Software and code

Policy information about availability of computer code

| | |
|---|---|
| Data collection | Reader Control Software TECAN Spark plate reader, MS Excel (Office 365),GROMACS 2024, DOCK3.7, EPU software (v.2.14.0) (Thermoscientific) |
| Data analysis | MS Excel (Office 365), GraphPad Prism 9.0, AmberTools 18 CPPTRAJ, getcontacts (https://getcontacts.github.io/), Chimera (v.1.16), Chimera (v1.17.3), UCSF ChimeraX (v.1.10), Coot 0.9.8.95, Phenix (1.21), CryoSPARC (v4.7.1), Relion-5.0, Topaz (v0.2.5a), PyMOL Molecular Graphics System (Schrödinger v 2.5.7) |

For manuscripts utilizing custom algorithms or software that are central to the research but not yet described in published literature, software must be made available to editors and reviewers. We strongly encourage code deposition in a community repository (e.g. GitHub). See the Nature Portfolio guidelines for submitting code & software for further information.

## Data

Policy information about availability of data

All manuscripts must include a data availability statement. This statement should provide the following information, where applicable:

- Accession codes, unique identifiers, or web links for publicly available datasets
- A description of any restrictions on data availability
- For clinical datasets or third party data, please ensure that the statement adheres to our policy

Data and materials availability: All relevant data generated and analyzed during this study are included in this article and its Supplementary Information. Source data and reporting summary are provided with this paper. CryoEM data are available as the PDB entry pdb_00009rhg (PDB ID 9RHG) (https://doi.org/10.2210/

pdb9RHG/pdb) and EMDB entry ID EMD-53969. All MD trajectories are deposited on GPCRmd (https://gpcrmd.org) with publication entry https://gpcrmd.org/dynadb/publications/1629/ and individual simulation IDs 2339 (docking pose), 2340 (\textit{apo} active), 2341 (structure pose) and 2364 (C476). Should any raw data files be needed in another format they are available from the corresponding author upon request. Expression vectors used and created for this work can be obtained from corresponding author.

## Research involving human participants, their data, or biological material

Policy information about studies with human participants or human data. See also policy information about sex, gender (identity/presentation), and sexual orientation and race, ethnicity and racism.

| | |
|---|---|
| Reporting on sex and gender | n.a. |
| Reporting on race, ethnicity, or other socially relevant groupings | n.a. |
| Population characteristics | n.a. |
| Recruitment | n.a. |
| Ethics oversight | n.a. |

Note that full information on the approval of the study protocol must also be provided in the manuscript.

# Field-specific reporting

Please select the one below that is the best fit for your research. If you are not sure, read the appropriate sections before making your selection.

- [x] Life sciences
- [ ] Behavioural & social sciences
- [ ] Ecological, evolutionary & environmental sciences

For a reference copy of the document with all sections, see nature.com/documents/nr-reporting-summary-flat.pdf

# Life sciences study design

All studies must disclose on these points even when the disclosure is negative.

| | |
|---|---|
| Sample size | No sample size calculation was performed. However, n > or = 3 is a typical sample size for biological experiments. Sample sizes including both number of independent experiments and technical replicates are stated in the respective figure legends. |
| Data exclusions | In general, no data were excluded. |
| Replication | At least three independent experiments were performed for each dataset found in the main text. The exact number of independent experiments and technical replicates is stated in the respective figure legends. |
| Randomization | Randomization was not relevant for our study, as it was purely based on cell-based experiments assessing ligand-induced or ligand-independent responses. These types of experiments do not necessarily require randomization. |
| Blinding | Blinding was not relevant for our study, as it was purely based on cell-based experiments. Analyzing these experiments is based on numerical data obtained from a plate reader and is therefore not subjective. |

# Reporting for specific materials, systems and methods

We require information from authors about some types of materials, experimental systems and methods used in many studies. Here, indicate whether each material, system or method listed is relevant to your study. If you are not sure if a list item applies to your research, read the appropriate section before selecting a response.

## Materials & experimental systems

| n/a | Involved in the study | |
|---|---|---|
| ☐ | ☒ | Antibodies |
| ☐ | ☒ | Eukaryotic cell lines |
| ☒ | ☐ | Palaeontology and archaeology |
| ☒ | ☐ | Animals and other organisms |
| ☒ | ☐ | Clinical data |
| ☒ | ☐ | Dual use research of concern |
| ☒ | ☐ | Plants |

## Methods

| n/a | Involved in the study | |
|---|---|---|
| ☒ | ☐ | ChIP-seq |
| ☒ | ☐ | Flow cytometry |
| ☒ | ☐ | MRI-based neuroimaging |

## Antibodies

| | |
|---|---|
| Antibodies used | N.A. |
| Validation | *Describe the validation of each primary antibody for the species and application, noting any validation statements on the manufacturer's website, relevant citations, antibody profiles in online databases, or data provided in the manuscript.* |

## Eukaryotic cell lines

Policy information about cell lines and Sex and Gender in Research

| | |
|---|---|
| Cell line source(s) | HEK293A cells: ThermoFisher Scientific (cat.-No.: R70507)<br>ΔFZD1-10 HEK293T cells were a kind gift from Benoit Vanhollebeke, Université de Bruxelles (PMID: 30026314) |
| Authentication | No |
| Mycoplasma contamination | Cell lines were regularly tested for mycoplasma contamination and found to be negative using the MycoStrip kit (InvivoGen) |
| Commonly misidentified lines (See ICLAC register) | None used. |

## Plants

| | |
|---|---|
| Seed stocks | N.A. |
| Novel plant genotypes | *Describe the methods by which all novel plant genotypes were produced. This includes those generated by transgenic approaches, gene editing, chemical/radiation-based mutagenesis and hybridization. For transgenic lines, describe the transformation method, the number of independent lines analyzed and the generation upon which experiments were performed. For gene-edited lines, describe the editor used, the endogenous sequence targeted for editing, the targeting guide RNA sequence (if applicable) and how the editor was applied.* |
| Authentication | *Describe any authentication procedures for each seed stock used or novel genotype generated. Describe any experiments used to assess the effect of a mutation and, where applicable, how potential secondary effects (e.g. second site T-DNA insertions, mosiacism, off-target gene editing) were examined.* |
